# Deletion of *Re*-citrate synthase allows for analysis of contributions of tricarboxylic acid cycle directionality to the growth of *Heliomicrobium modesticaldum*

**DOI:** 10.1128/aem.01772-24

**Published:** 2025-03-06

**Authors:** Alexandria M. Layton, Christopher McCauley, Kevin E. Redding

**Affiliations:** 1 School of Molecular Sciences, Arizona State University 200398 , Tempe, Arizona, USA; 2 Center for Bioenergy and Photosynthesis, Arizona State University 7864 , Tempe, Arizona, USA; 3 Arizona Department of Health Services 6577 , Phoenix, Arizona, USA; University of Nebraska-Lincoln, Lincoln, Nebraska, USA

**Keywords:** heliobacteria, citrate synthase, TCA cycle, electron source, acetate metabolism

## Abstract

*Heliomicrobium modesticaldum,* a phototrophic member of the phylum Firmicutes and family Clostridiales, possesses most of the enzymes specific to the reductive tricarboxylic acid (rTCA) cycle, except for the key enzyme, ATP-citrate lyase. It is thought to utilize a split TCA cycle when growing on pyruvate as a carbon source, in which the oxidative TCA (oTCA) direction generates most of the 2-ketoglutarate, but some can be produced in the reductive direction. Although a typical *Si*-citrate synthase gene is not found in the genome, it was suggested that gene HM1_2993, annotated as homocitrate synthase, actually encodes *Re*-citrate synthase, which would function as the initial enzyme of the oTCA cycle. We deleted this gene to test this hypothesis and, if true, see what effect severing access to the oTCA cycle would have on this organism. The endogenous CRISPR-Cas system was used to replace the open reading frame with a selectable marker. The deletion mutants could grow on pyruvate but were unable to grow phototrophically on acetate + CO_2_ as carbon source. Growth on acetate could be rescued by the addition of different electron sources (formate or ascorbate), suggesting that the oTCA cycle is used to oxidize acetate to generate electrons required to drive the carboxylation of acetyl-CoA. The deletion mutants were capable of growing in acetate minimal media without additional organic supplements beyond formate, demonstrating that the rTCA cycle can be employed to support sufficient 2-ketoglutarate production in this organism, unlike citrate synthase mutants in several chemoheterotrophic organisms utilizing the oTCA cycle.

**IMPORTANCE:**

Heliobacteria are a unique group of phototrophic bacteria that are obligate anaerobes and possess a rudimentary system to use light as a source of energy. They do not make oxygen or fix carbon dioxide. Here, we explore their fundamental carbon metabolism to understand the role and operation of the central TCA cycle. This work shows both the role and operation of this cycle under different growth modes and explains how these organisms can obtain electrons to drive their biosynthetic metabolism. This foundational knowledge will be crucial in the future when attempts are made to use this organism as a platform for oxygen-sensitive synthesis of compounds in an anaerobe that can use light as its energy source.

## INTRODUCTION

The Heliobacteriaceae are an anaerobic, gram-positive family of bacteria, comprising the only phototrophic group in the phylum Firmicutes. Originally found in simple garden soil (1, 2), they have since also been found in volcanic soil, rice paddies, soda lakes, and hot springs (3) and are split into two groups, an alkaliphilic group growing at high pH (pH 8–9.5), and a neutrophilic group (pH 6–8) (2). Despite occupying different niches, all members display similar aspects of energy, carbon, and electron metabolism. They are capable of growing phototrophically, utilizing a homodimeric reaction center, and members of the neutrophilic group are capable of growing chemotrophically in the dark through fermenting a carbon source such as pyruvate (2, 4–6). Unlike other species of anaerobic phototrophs, the heliobacteria are obligate heterotrophs. Furthermore, it has been inferred that they are organotrophs, as no inorganic electron donor is required for growth, thus implying that they obtain electrons from their carbon source, intertwining carbon and electron metabolism (5, 7, 8).

**Fig 1:**
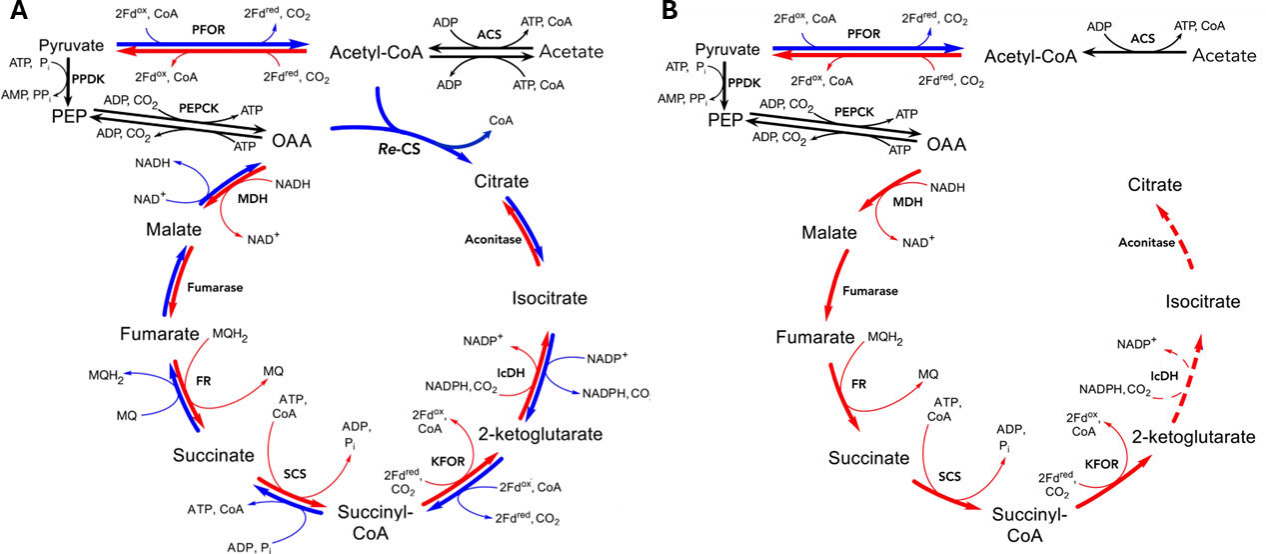
Proposed TCA cycle-based carbon metabolism employed by *H. modesticaldum* in WT cells (**A**) and the mutant lacking CS (**B**). (**A**) The TCA cycle is split, employing both the forward (oTCA) and the reverse (rTCA) cycle directions to generate 2- KG. Genome annotations describe the presence of rTCA cycle-specific enzymes fumarate reductase (FR) and 2-ketoglutarate:ferredoxin oxidoreductase (KFOR), as well as an NADP-dependent isocitrate dehydrogenase (IcDH). *H. modesticaldum* can potentially perform the entire oTCA cycle, considering that all enzymes (with the exception of *Re*-citrate synthase) are reversible. (**B**) The proposed remnant rTCA cycle created by the loss of citrate synthase. Access to the oTCA cycle is severed, leaving only access to the rTCA cycle portion of the cycle. Dashed lines indicate uncertainty about the flow of metabolites through that step. Enzyme abbreviations are as follows: PFOR, pyruvate:ferredoxin oxidoreductase; ACS, acetyl-CoA synthetase; PPDK, pyruvate, phosphate dikinase; PEPCK, phosphoenolpyruvate carboxykinase; MDH, malate dehydrogenase; FR, fumarate reductase; SCS, succinyl-CoA synthetase; KFOR, 2-oxoglutarate:ferredoxin oxidoreductase; IcDH, isocitrate dehydrogenase; and *Re*-CS, Re-citrate synthase.

*Heliomicrobium modesticaldum* is a moderately thermophilic member of the Heliobacteriaceae. Although able to use phototrophy to produce ATP, they rely on organic carbon as their source of carbon and their primary source of electrons as well (9). *H. modesticaldum*, like the other members of the Heliobacteriaceae, is hypothesized to use a split tricarboxylic acid (TCA) cycle (7) (Fig. 1A). Its genome contains genes for all the rTCA cycle enzymes except ATP-citrate lyase (10, 11). It uses a menaquinone-dependent fumarate reductase in place of the more common succinate dehydrogenase, and the version of isocitrate dehydrogenase that is specific for NADP rather than NAD. In addition, the heliobacteria employ the ferredoxin-dependent versions of pyruvate and 2-ketoglutarate dehydrogenase (10, 12), known as pyruvate:ferredoxin oxidoreductase (PFOR) and 2-ketoglutarate:ferredoxin oxidoreductase (KFOR), respectively. Isotopic analysis confirms that both the oxidative TCA (oTCA) cycle and reverse (rTCA) cycle contribute to the generation of 2-KG (7). Although flux was found to be largely through the oTCA cycle, the partial rTCA cycle accessible to *H. modesticaldum* does function at low levels when grown on pyruvate. As with all heliobacteria, *H. modesticaldum* is obligately heterotrophic, as its partial rTCA cycle is unable to support autotrophic growth due to the lack of ATP-citrate lyase. However, some CO_2_ is fixed via anaplerotic reactions (7).

**Fig 2:**
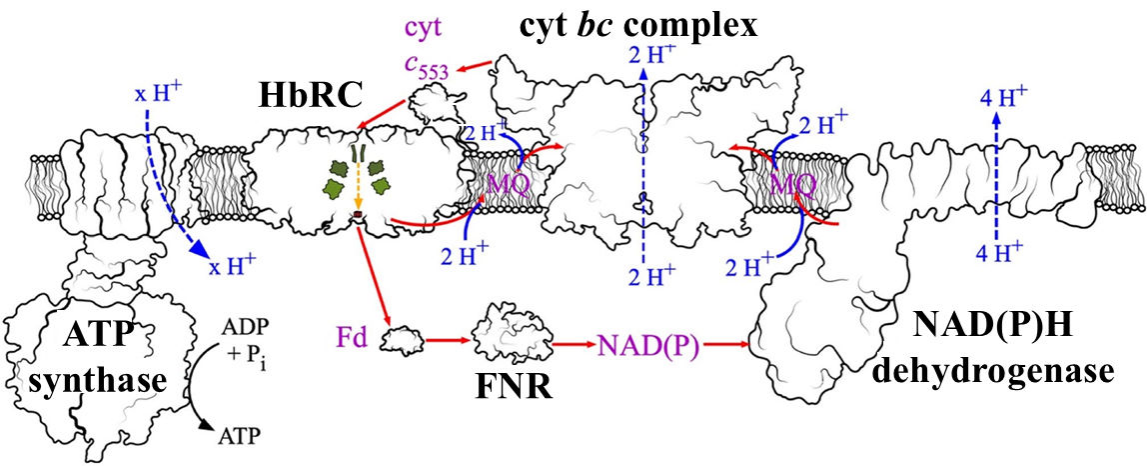
Electron flow schematic of *H. modesticaldum* (13). Actual structures of the related enzymes are displayed in the context of a lipid bilayer, with the enzyme names displayed in black (ATP synthase, HbRC, cyt *bc* complex, NAD(P)H dehydrogenase, FNR). Cofactors and molecules necessary for maintaining the redox pool are shown in purple (Fd, NAD(P), cyt *c*_553_, MQ). Proton transfer events are labeled with blue, dashed arrows, and electron transfer events are labeled with red, solid arrows.

The light-driven electron transport pathway of heliobacteria is shown in Fig. 2 (13). The heliobacterial reaction center (HbRC) is used to reduce the cytosolic ferredoxin (Fd) pool (12) and oxidize periplasmic cytochrome (Cyt) *c*. The redox gradient thus created drives overall electron transport through the NADP and menaquinone (MQ) pools, via a Fd:NADP+ oxidoreductase (FNR) and NADPH dehydrogenase, respectively. The cytochrome *bc* complex closes the loop by passing electrons from the MQ pool back to the Cyt *c* pool (13). Proton pumping by the last two complexes creates a proton electrochemical gradient that can be used to drive ADP phosphorylation by ATP synthase. An important point to highlight here is that this cycle provides a valuable service to cellular metabolism: interconversion and upconversion of reducing equivalents. Electrons can be fed into the cycle at any point (Cyt *c*, MQ, NADH, NADPH, or Fd) and removed from the cycle at any point. Thus, two reducing equivalents produced by the oTCA cycle in the form of NADPH (by isocitrate dehydrogenase), two Fd^red^ (by KFOR), MQH_2_ (by fumarate reductase), or NADH (by malate dehydrogenase) can be converted to two reducing equivalents at any other level, including the lowest potential (highest energy) electron carrier, Fd.

Despite the absence of an identified citrate synthase (CS) gene, citrate synthase activity was detected in *H. modesticaldum*, but it was O_2_-sensitive; the results of isotopic-labeling experiments were consistent with this species making use of a *Re*-CS rather than the more common *Si*-CS (7). The oxygen-sensitive *Re*-CS has been found to be utilized by other anaerobic bacteria, spanning the Firmicutes (14–18), *Chloroflexota* (19, 20), and *Thermodesulfobacteriota* (21, 22) phyla, as well as an archaeon, *Ignicoccus* (23). Although *Re*-CS and S*i*-CS perform the same overall reaction, *Re*-CS is entirely unrelated to *Si*-CS and is instead related to homocitrate synthase (24); this has resulted in the gene being misannotated as homocitrate synthase or isopropylmalate synthase in several genomes of the previously mentioned phyla (19, 21). The gene HM1_2993, annotated in the *H. modesticaldum* genome as homocitrate synthase, was previously proposed to be the *Re*-CS gene (7) but this has not been confirmed. Here, we aimed to delete this gene to test its identity as the gene *Re*-CS and analyze its effects on the cell’s metabolism and growth.

Citrate synthase can be considered the entrance point into the oTCA cycle. Without it, heliobacteria should be unable to use the oTCA cycle (Fig. 1B). The effect of removing *Re*-CS on the functioning of an organism has not been analyzed in any species it has been found in. However, deletion of genes for the analogous enzyme, *Si*-CS, in the proteobacteria *Escherichia coli* (25) and rhizobium *Ensifer meliloti* (26, 27) resulted in strains that required supplemental glutamate or arabinose. Judging from the analysis of other Firmicutes employing *Re*-CS, one would predict a requirement for glutamate to be observed in *Re*-CS deletion mutants for this group. Isotope analysis of *Clostridium thermocellum*, which contains both *Si*-CS and *Re*-CS, displays a complete lack of metabolic flow to 2-KG through the rTCA pathway (18). Similarly, it was suggested that *Clostridium acetobutylicum* does not exchange carbon between fumarate and 2-KG (28) in the rTCA direction (17). In addition, the TCA cycle appears to be incomplete, lacking malate dehydrogenase and any enzymes to convert 2-KG to succinate, in another *Re*-CS employer, *Desulfovibrio vulgaris* (22). This implies that many organisms in the Clostridiales would have a requirement for supplemental glutamate or glutamine to sustain the production of amino acids originating from 2-KG (i.e., Glu, Gln, Pro, and Arg) if CS were absent. Conversely, it has been found that *H. modesticaldum* is capable of performing the rTCA cycle to produce some 2-KG (7), and thus could potentially sustain 2-KG production even without a supplemental source in the absence of *Re*-CS activity.

We have used *H. modesticaldum* extensively as a model organism for phototrophy in a simple phototroph (13, 29–32), but we have only scratched the surface in studying other aspects of heliobacterial metabolism (12, 33, 34). We wish to expand the use of this organism by modifying its metabolism. Therefore, we must first understand its basic metabolic capabilities and limitations, especially in carbon and electron metabolism. Using the tools we have developed to edit the chromosome (35), we have removed the key access point to the oTCA cycle, which will allow us to answer questions about the purpose of the oTCA cycle in this organism, considering its redundancy with the partial rTCA cycle that it possesses.

## MATERIALS AND METHODS

### Strains and culture conditions

*Escherichia coli* strains (Top 10 during plasmid construction and harvesting, s17-1 during conjugation with *H. modesticaldum*) were grown in Luria-Bertani (LB) broth (BD Difco, Franklin Lakes, NJ). When necessary, antibiotics erythromycin (Sigma-Aldrich, St. Louis, MO, 100 µg/mL), chloramphenicol (Acros, Geel, BE, 15 µg/mL), and kanamycin (GoldBio, St. Louis, MO, 50 µg/mL) were added to the media. Cultures were grown at 37°C overnight with shaking at 250 rpm.

*H. modesticaldum* strains were grown and handled in a Coy anaerobic chamber (Coy Laboratory Products, Grass Lake, MI), filled with an atmosphere of 20% CO_2_, 2%–4% H_2_, and balanced N_2_. Four strains of *H. modesticaldum* were grown: wild-type ICE1 (WT), and three citrate synthase knockout strains (95 R-6, 95 R-11, and 95 R-12), in a variety of media, which are listed in Table 1. All cultures were grown at 50°C under 790-nm light-emitting diodes (LEDs) (Marubeni America, New York, NY) at a flux of 30 µmol photons m^-2^ s^-1^. Antibiotics erythromycin (10 µg/mL) and kanamycin (50 µg/mL) were added to plates and liquid cultures when necessary. Several supplements were added to the media throughout the study. Stocks of the supplements used, sodium formate (Alfa Aesar, Haverhill, MA, 30 mM final), sodium ascorbate (Sigma-Aldrich, 20 mM final), indigo trisulfonate potassium salt (ITS, Riedel-De Haen, now Honeywell, Charlotte, NC, 0.01 mM final), and L-glutamine (Sigma-Aldrich, 10 mM final), were prepared using N_2_-bubbled water in an anoxic glovebox, filter sterilized, and added to autoclaved media at the indicated concentration.

**TABLE 1:** Media types used throughout this study

Media name	Carbon source	Media base	Source
PYE	20 mM sodium pyruvate	4 g/L yeast extract (Thermo Scientific, Waltham, MA)	(9)
ABYE	20 mM sodium acetate 10 mM sodium bicarbonate	4 g/L yeast extract	This paper
ABYE/4	20 mM sodium acetate 10 mM sodium bicarbonate	1 g/L yeast extract	This paper
PMS	20 mM sodium pyruvate	Minimal salts	(4, 9)
BMS	10 mM sodium bicarbonate	Minimal salts	(4)
ABMS	20 mM sodium acetate 10 mM sodium bicarbonate	Minimal salts	(4)

### Plasmid construction and knockout generation

**Fig 3:**
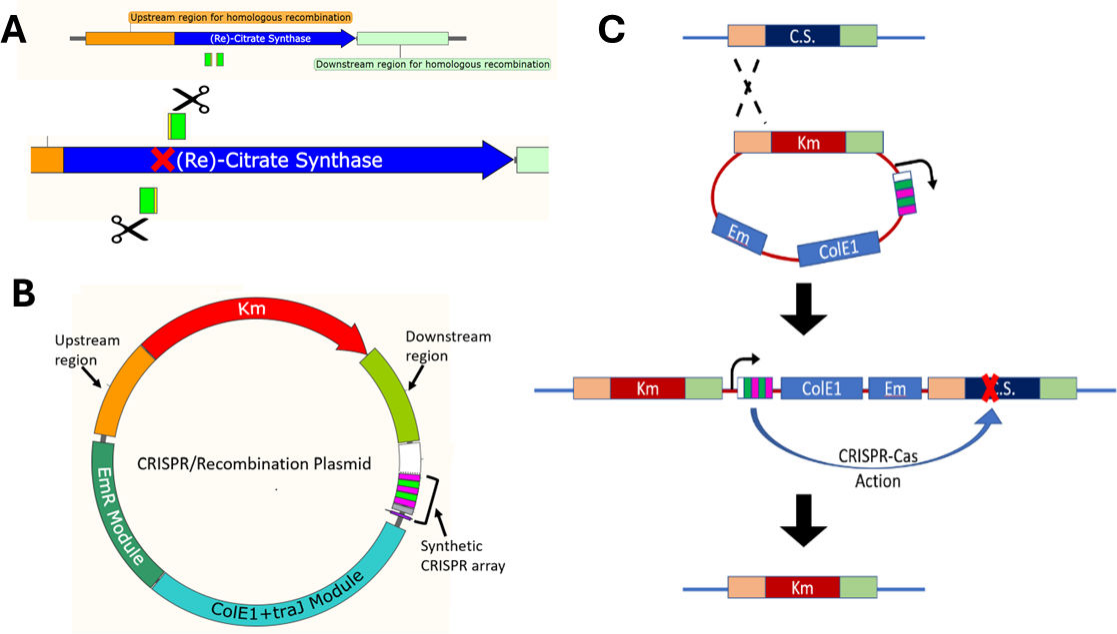
Genetic tools and processes to obtain the citrate synthase knockout. (**A**) Schematic of HM1_2993 on the chromosome in its entirety (top), along with the upstream (orange) and downstream (mint green) regions used to construct the homologous recombination cassette, and zoomed in (bottom) to show the selected spacer sequences (green) that were inserted into the synthetic CRISPR array; scissors indicate directionality of Cas endonuclease action after targeting to the site. (**B**) Non-replicating plasmid containing the recombination cassette featuring the upstream and downstream regions flanking a kanamycin resistance gene (*aph3*). Also included is the synthetic CRISPR array, containing the spacers targeting the native citrate synthase (green) between repeat sequences specific to *H. modesticaldum* (magenta). (**C**) Schematic of the envisioned process. Homologous recombination between a flanking sequence in the plasmid and chromosome results in integration of the plasmid into the chromosome and kanamycin resistance. Action of the CRISPR-Cas system will result in targeting of the HM1_2993 loci in integrant (and wild-type) chromosomes, with only the rare double recombination event that deletes HM1_2993 being resistant to the action of the CRISPR/Cas system.

All oligonucleotides were purchased from Integrated DNA Technologies (IDT) (Coralville, IA) and are listed in Table 2. The non-replicating vector containing a synthetic CRISPR array, pPB1213, was used as the backbone for assembling the plasmid used to generate the citrate synthase knockout strain. Using the method described previously (35), artificial spacers targeting the citrate synthase gene were assembled by annealing oligonucleotides containing the spacer sequences (CitSyn Spacer 1 for/rev, Spacer 7 for/rev) and the CRISPR repeat sequence (CRISPR repeat for/rev) and then ligated into the synthetic CRISPR array in pPB1213 using BsaI (a type IIs restriction enzyme) during a Golden Gate ligation process, generating pAL95. The template for homologous recombination was assembled by amplifying genomic regions upstream and downstream of HM1_2993, amplifying the Km^r^ gene (*aph3*) controlled by the cbp_2 promoter from pPB1109, digesting the amplified pieces with the appropriate enzymes (HindIII/XbaI on the upstream region, NheI/BamHI on the cbp_2-*aph3* cassette, and BamHI/HindIII on the downstream region), and ligating them together and into the HindIII site on pAL95, generating pAL95R as seen in Fig. 3. All plasmids and descriptions are found in Table 3.

**TABLE 2:** List of primers used throughout this study^*a*^

Oligonucleotide	Sequence (5’−3’)	Purpose
CitSyn Spacer 1 for	AAAGCTGTGACGCTGTCGCCATCTCCATCTCCACTTCCGACGT	To assemble fake spacers targeting HM1_2993 on genome
CitSyn Spacer 1 rev	TGAAACGTCGGAAGTGGAGATGGAGATGGCGACAGCGTCACAG	
Spacer 7 for	TTGAAAGGGTTCCAACCCATGATGCTGGCCTTCAGGCCCATCTT	
Spacer 7 rev	AAACAAGATGGGCCTGAAGGCCAGCATCATGGGTTGGAACCCTT	
CRISPR repeat for	TTCACTGGATGACCCGCTATGTAGGGGA	To assemble the repeat sequence of *H. modesticaldum*’s CRISPR array to assemble 2-spacer 3-repeat synthetic array
CRISPR repeat rev	TCAATCCCCTACATAGCGGGTCATCCAG	
CitSyn_up_for_HindIII	TGCCAAGCTTATTCAGCAGATCTATCGCAAGG	Amplify 500 bp sequence matching genomic DNA upstream of HM1_2993
CitSyn_up_rev_XbaI	TTCTCTAGACCTCACCCCTCTAAAGTATTTTTTC	
CitSyn_down_for_BamHI	TTCTGGATCCTGCACCGGCATGATTTGTTGG	Amplify 500 bp sequence matching genomic DNA downstream of HM1_2993
CitSyn_down_rev_HindIII	GGTGAAGCTTTAAGGGTGGATGCGGTGAACG	
cbp_2 NheI for	TAATGCTAGCGAGTCGTGACTAAGAACGTCAA	Amplify cbp_2-*aph3* from pPB1109 to insert between two homologous region sequences amplified from genome
KI rev BamHI	TTCTGGATCCCTAAAACAATTCATCCAGTAAAATATAATATTTTATTTTCTCC	
CS short BamHI for	ATTTGGATCCGTGTTGAACAACCGGCAAATTACC	Amplify HM1_2993 from genomic DNA to add as a complement to knockout strains
Cit Syn XhoI rev	ATCCACTCGAGGCTCAGTGTTCGGGATACC	
PgapDH PspOMI for	TAATACGGGCCCTAATTACTGTATCTCTCTGGC	Amplify the gapDH promoter to add individually to pMTL86251
PgapDH BamHI rev	TAATACGGATCCTAATATCGCCTCCTATTGTAAATTAAA	
CitSyn Probe for	AAAAGTCTATCGGTATCGAGC	Probe section of genomic DNA to analyze if the original gene or Km is inserted
CitSyn Probe rev	CAAAAAGGCGAAGTTCAGG	

^*a*^ All primers were ordered from IDT.

**TABLE 3:** List of plasmids used in this study

Plasmid	Description	Source
pMTL86251	Modular shuttle vector for use in clostridial bacteria	(36)
pPB1213	Non-replicating plasmid based on pMTL8025C containing backbone for synthetic CRISPR array	(35)
pPB1109	Plasmid used during the production of ΔpshA *H. modesticaldum* through previous study, containing a synthetic array to delete *pshA* from the genome as well as cbp_2 + *aph3* cassette.	(35)
pAL95	pPB1213 backbone with spacers targeting HM1_2993 in synthetic CRISPR array	This paper
pAL95R	pAL95 with homologous recombination region to replace HM1_2993 on the genome inserted into HindIII sites	This paper
pGapDH	pMTL86251 with clostridial promoter gapDH inserted into NotI/BglII sites	(37) This paper
pALCS	pGapDH with original HM1_2993 amplified from *H. modesticaldum* genome inserted behind GapDH in BglII/XhoI sites	This paper

Plasmid pAL95R was introduced into WT *H. modesticaldum* through conjugation methods previously described (38), selecting on PYE medium supplemented with 10 mM glutamine as the nitrogen source (instead of the typical 7 mM ammonium chloride [9]), with both erythromycin (10 µg/mL) and kanamycin (50 µg/mL). Strains were assessed for Km^r^ and Em^r^; loss of Em^r^ with Km^r^ retention indicated a full recombination event and loss of the plasmid. Genomic DNA from Em-sensitive and Km-resistant strains was prepared using the NucleoSpin Microbial DNA kit (Macherey-Nagel, Duren, Germany). DNA was amplified using two sets of primers: probe (CitSyn Probe for/rev) and integration (CitSyn_up_for_HindIII and CitSyn_down_rev_HindIII). Probe primers amplified the entire genomic DNA region for replacement of HM1_2993 with cbp_2-*aph3*, whereas integration primers could detect retention of the plasmid in the genome. Amplified products were further digested with EcoRV to easily visualize the differences in product sizes reflective of HM1_2993 (2384 bp, digested to 1178 bp, and 1206 bp) or cbp_2-*aph3*Km^r^ (2670 bp, digested to 1891 bp, and 688 bp). PCR products were further analyzed using Sanger sequencing.

### Complementation plasmid construction

The strong promoter gapDH (37) was amplified using primers (PgapDH PspOMI for, PgapDH BamHI rev), digested with restriction enzymes PspOMI and BamHI, and ligated into pMTL86251 using the NotI and BamHI sites, generating plasmid pGapDH. HM1_2993 was amplified from genomic DNA using primers (CS short BamHI for, Cit Syn XhoI rev), digested with restriction enzymes BamHI and XhoI, then inserted into pGapDH into the BamHI and XhoI sites, generating the complementation plasmid pALCS. Plasmids were introduced to *H. modesticaldum* ΔHM1_2993 strains utilizing conjugation methods previously outlined (38) using PYE media supplemented with 10 mM glutamine.

### Growth assays

Growth assays were set up either in 96-well plates and read using an Epoch Microplate spectrophotometer (BioTek Instruments, Inc., Winooski, VT) or performed in aluminum crimp-sealed vials with media bubbled with either 80% N_2_/20% CO_2_ gas or 75% N_2_/20% CO_2_/5% H_2_ gas (media were bubbled prior to inoculation). When using sealed vials, inoculations and removal of media for readings were performed using needles and syringes to preserve the atmosphere of the vials (reflecting which gas type was used for bubbling), and media were transferred to plates to be read by the Epoch spectrophotometer. Growth data were fit to the logistic equation as described previously (13).

## RESULTS

### Similarity of HM1_2993 to known *Re*-citrate synthases

The HM1_2993 gene was originally annotated as a homocitrate synthase. It seems isolated in the genome, surrounded by several genes for hypothetical proteins without appearing to be part of an operon. Interestingly, the best match to a bona fide homocitrate synthase in the *H. modesticaldum* genome is *nifV*, which is part of the nitrogen fixation gene cluster. This makes perfect sense, as homocitrate is part of the Fe-Mo cofactor in dinitrogenase but typically plays no role in metabolism otherwise. The gene for homocitrate synthase is frequently a part of the *nif* operon and is only expressed during nitrogen-fixation conditions, as seen in *H. modesticaldum* (33). Thus, this organism should have no need for a second homocitrate synthase gene, suggesting that HM1_2993 plays another role.

**Fig 4:**
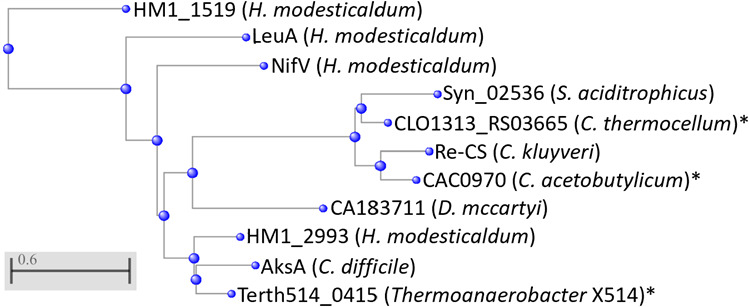
Phylogenetic tree generated by the COBALT constraint-based multiple alignment tool from NIH comparing known and putative (indicated by “*”) *Re*-citrate synthase protein sequences, as well as related genes. Sequence identifiers or enzyme names (if designated) are displayed with the host species name in parentheses. All protein sequences are listed in Table S1, and all percent identities are listed in Fig. S1.

Having found that *H. modesticaldum* used a *Re*-CS in the oTCA cycle, Tang et al. (7) proposed that this enzyme might be encoded by HM1_2993, as *Re*-CS is evolutionarily related to homocitrate synthase. When the predicted protein sequence of HM1_2993 is aligned to known *Re*-CS sequences from *Syntrophus aciditrophicus* and *Clostridium kluyveri*, the percent identity is relatively low, at around 28% for both (Fig. S1). Comparison of HM1_2993 to putative *Re*-CS genes from two other clostridial species, *C. acetobutylicum* and *C. thermocellum*, yielded similar identities (27% and 29%, respectively). However, when comparing HM1_2993 to confirmed *Re*-CS genes from *Dehalococcoides mccartyi* 195 and *Clostridioides difficile*, and the putative gene from *Thermoanaerobacter sp*. X514, the identity is about 36%, 62%, and 68%, respectively (Fig. S1). A phylogenetic tree generated using the NIH COBALT tool (Fig. 4) clusters the HM1_2993 gene with the *Re*-CS genes from *Thermoanaerobacter* and *Clostridioides,* but distinctly from those from *Syntrophus* and *Clostridium*. However, all of these genes are quite distinct from the other heliobacterial genes that could potentially be the *Re*-CS, annotated as homocitrate synthase (*nifV*), isopropylmalate synthase (*leuA*), or citramalate synthase (HM1_1519).

### Creation of HM1_2993 gene deletion strain

To delete the HM1_2993 gene, a homologous recombination cassette was provided to replace HM1_2993 with a kanamycin-resistance gene (*aph3*), whereas simultaneously the endogenous CRISPR/Cas system was programmed to target DNA sequences within HM1_2993 by expressing artificial CRISPR guide RNAs. These elements were introduced into *H. modesticaldum* on a non-replicating plasmid (Fig. 3). The action of the CRISPR/Cas system and homologous recombination should lead to replacement of the HM1_2993 locus on the chromosome with *aph3*, resulting in kanamycin-resistant cells lacking HM1_2993.

**Fig 5:**
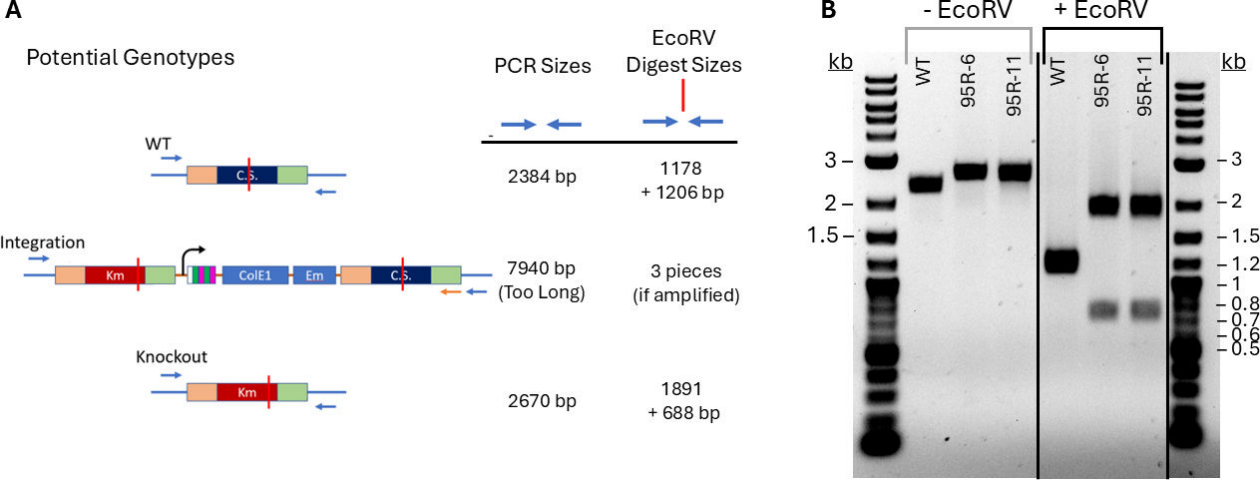
(**A**) Set-up and expectations of PCR/digest of genomic DNA to probe the presence of HM1_2993 (blue, “CS”) or *aph3* (red, “Km”). Probe primers (blue arrows) anneal to chromosomal DNA but not to any sequences in plasmid pAL95R. Diagrams of the three potential genotypes (“WT,” “Integration,” and “Knockout”) are provided to the left. Note that the plasmid can integrate in two different orientations, but only one is shown. Locations of EcoRV sites are indicated by red vertical lines. On the right, the expected sizes of the PCR products for each genotype are provided, and expected sizes of the PCR products when they are subsequently digested with EcoRV. (**B**) Agarose gel of the PCR products obtained using genomic DNA from WT and the two putative knockout strains (95R-6, 95R-11) as templates. PCR products were generated using “probe” primers and are shown on the left (-EcoRV). The right side shows the products after digestion with EcoRV (+EcoRV). (Cropped gel image shown; full image of the gel, also including other PCR tests, can be found as Fig. S2.)

Previous studies indicated that *H. modesticaldum* can use glutamine as a nitrogen source (9). Throughout the conjugation and genetic testing stages, strains were grown on PYE medium with 10 mM glutamine as the nitrogen source (in the place of the typical 7 mM ammonium) to encourage glutamine uptake. Glutamate is not directly utilized by *H. modesticaldum* as either a nitrogen or carbon source (9); hence, utilizing glutamine as a nitrogen source would result in the production of glutamate, which could be further used to support production of the Glu family of amino acids in the event of citrate synthase deletion. Transformation resulted in the production of kanamycin-resistant colonies that were further assessed for erythromycin resistance. The erythromycin-resistance gene on the plasmid should be removed by a double-recombination event (Fig. 3C); thus, erythromycin resistance would indicate the presence of an integrated plasmid. Those that were kanamycin-resistant and erythromycin-sensitive are candidates for having the desired genetic modification. Genomic DNA was prepared from several such strains and analyzed by PCR, restriction digest, and DNA sequencing to test for gene replacement. Figure 5A shows the expected sizes of the PCR reaction using “probe” primers. The “probe” reaction examines the chromosomal locus using primers annealing to sequences outside the flanking regions included on the plasmid. The predicted sizes of the PCR products from HM1_2993 and *aph3* are similar: 2384 and 2670 bp. Therefore, digestion with EcoRV was used to distinguish them, as there is a single EcoRV site in HM1_2993 and *aph3* but at different positions (Fig. 5A), resulting in different band patterns after digestion: 1178 + 1206 bp for HM1_2993 and 1891 + 688 bp for *aph3*. For an integrated plasmid, the “Probe” reaction products would likely not be discernible due to limitations of the PCR (i.e., the amplicon is too large).

As shown in Fig. 5B, the two putative knockout strains (95R-6, 95R-11) display the band pattern expected for the knockout genotype, and there are no additional bands matching those expected for the WT genotype. Shown are both the purified products from the PCR reactions (-EcoRV), displaying the slight variation between the WT and knockout genotypes (2384 bp and 2670 bp, respectively), as well as the products from digestion with EcoRV (+EcoRV). Both PCR and digest suggest that the two putative knockouts have indeed performed a double recombination event in which the citrate synthase has been replaced with the kanamycin resistance gene. The sequences of the PCR products using the “Probe” primer set were obtained using Sanger sequencing. The sequence of the amplicon from the knockout strains confirmed replacement of the HM1_2993 gene with the kanamycin-resistance gene and was exactly as designed at the nucleotide level (Fig. S3).

### Characterization of HM1_2993 gene deletion strains

With several independent knockout strains obtained, we turned to analyze the phenotype of the ΔHM1_2993 deletion. First, we examined the mutants’ ability to grow phototrophically on the two major carbon sources (pyruvate or acetate). It has previously been demonstrated that growth of heliobacteria on acetate requires light and the presence of CO_2_ (7). This can be explained by the fact that conversion of acetate to acetyl-CoA requires ATP and that acetate cannot be fermented to generate ATP; instead, phototrophic cyclic electron transport must be used to produce ATP (Fig. 2). Conversion of acetate (as acetyl-CoA) to pyruvate by PFOR also requires CO_2_, as do other important carboxylation reactions, such as the one catalyzed by phosphoenolpyruvate carboxykinase PEPCK. In addition, reduced ferredoxin is required to drive the PFOR reaction in the direction of pyruvate (Fig. 1).

In preliminary experiments, we found that WT *H. modesticaldum* does not require H_2_ in the headspace in order to grow phototrophically on acetate + CO_2_ (Fig. S4). Thus, the only source of electrons available to cells grown on acetate is the acetate itself, and the only method available to oxidize acetate is the oTCA cycle. Thus, we predict that a portion of the acetate imported into the cell (roughly 20-25%) would be oxidized fully to CO_2_ by the action of the oTCA cycle to provide electrons for anabolic metabolism (especially the PFOR reaction), and furthermore, that a loss of citrate synthase would block entry of acetyl-CoA into the oTCA cycle and inhibit growth on acetate. In contrast, growth on pyruvate, which can be oxidized directly by PFOR, should not require CS and should not be impacted by the loss of *Re*-CS.

To test these hypotheses, WT and ΔHM1_2993 strains were grown in a standard medium containing yeast extract (YE) and either pyruvate or acetate + bicarbonate as carbon sources (PYE or ABYE, respectively). All strains grew similarly in PYE under illumination, displaying similar growth rates and maximal OD_735_ values (Fig. S5). In ABYE, WT grows to a similar maximal OD_735_ as when grown in PYE. However, in ABYE, the ΔHM1_2993 strains attained maximal cell densities roughly half of that in PYE, although the growth rates are similar in the two growth conditions. The estimated rates for all strains range between 0.15 h^−1^ and 0.22 h^−1^, with the exception of ABYE-grown WT, with a rate closer to 0.4 h^−1^ in this particular test.

It has been shown previously that yeast extract can support limited growth of *H. modesticaldum* (8), which could account for the limited growth of the CSKO mutants on ABYE. We were initially unable to grow these mutants in minimal medium on acetate + CO_2_ (but this could be remedied later, see below). Therefore, we reduced the amount of yeast extract 4-fold in the second test, which was previously shown to limit the impact of carbon/electron sources from the YE but still stimulate growth, perhaps due to the presence of vitamins or amino acids (7).

**Fig 6:**
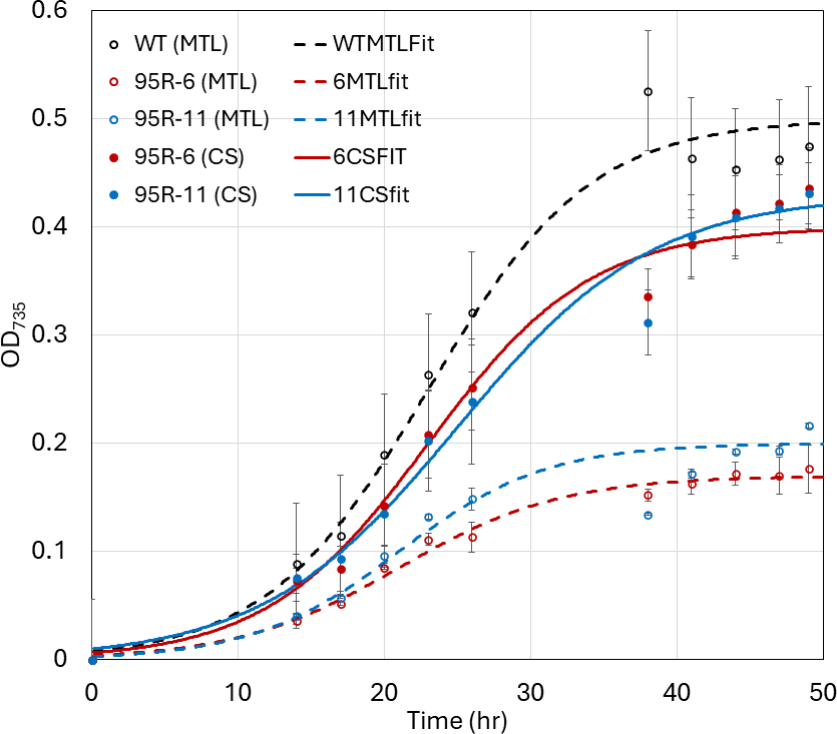
Effect of complementation of HM1_2993 in ΔHM1_2993 strains (95R-6, red; 95R-11, blue) compared with growth of WT (black). Strains were grown in ABYE/4 media (with 10 µg/mL Em) in open 96-well plates with continual access to the H_2_/N_2_/CO_2_ atmosphere. Closed circles and solid lines follow the growth of strains transformed with a plasmid containing a copy of HM1_2993 (CS) under the control of the strong *gapDH* promoter, whereas open circles and dashed lines show the growth of strains transformed with empty vector (MTL). Data points represent the average of three separately grown replicates of each strain, and error bars represent the standard deviation (*n* = 3). Lines represent the fitting of the points to a logistic equation.

Using the medium with reduced YE (ABYE/4), we tested the hypothesis that the negative effect in acetate growth displayed by the mutants was truly due to the loss of HM1_2993. Upon introduction of a plasmid providing expression of HM1_2993, this negative effect was largely remediated (Fig. 6). Based on these results, we conclude that loss of HM1_2993 is responsible for the poor growth on acetate as a carbon source. Furthermore, we hypothesize that HM1_2993 does indeed encode a *Re*-CS that is used in the oTCA cycle in heliobacteria and that the reason for poor growth on acetate is due to the cells being limited for reducing equivalents due to the loss of oTCA cycle function.

### Testing the use of different electron sources for growth on acetate

Our hypothesis is that the ΔHM1_2993 mutants’ poor growth on acetate is due to electron limitation. This hypothesis predicts that it would be possible to restore growth on acetate by the addition of alternate electron sources that are independent of the TCA cycle. We tested this by using three very different electron donors in medium containing lowered YE (ABYE/4) to see if we could improve phototrophic growth on acetate + CO_2_.

We first attempted to use hydrogen as an electron source, as it can be used by many anaerobic bacteria. The genome of *H. modesticaldum* contains genes predicted to encode a membrane-bound [NiFe]-hydrogenase that oxidizes H_2_ and reduces menaquinone (overall reaction: H_2_ + MQ → MQH_2_) (2, 10). Thus, in concert with the phototrophic cyclic electron flow pathway, these electrons could be ferried to Fd, to be used by PFOR (and other enzymes). However, the activity of this enzyme had never been tested in heliobacteria. As the atmosphere of the anaerobic chamber contains N_2_ (~77%), CO_2_ (~20%), and H_2_ (~2-3%), the headspace of a culture bottle will initially contain 2%–3% H_2_. We performed a simple experiment to monitor H_2_ in the headspace of WT cultures growing in PYE medium and found that most of the H_2_ disappearance occurred as growth was slowing (Fig. S6). In follow-up experiments (data not shown), we found that the onset and extent of H_2_ uptake were variable.

**Fig 7:**
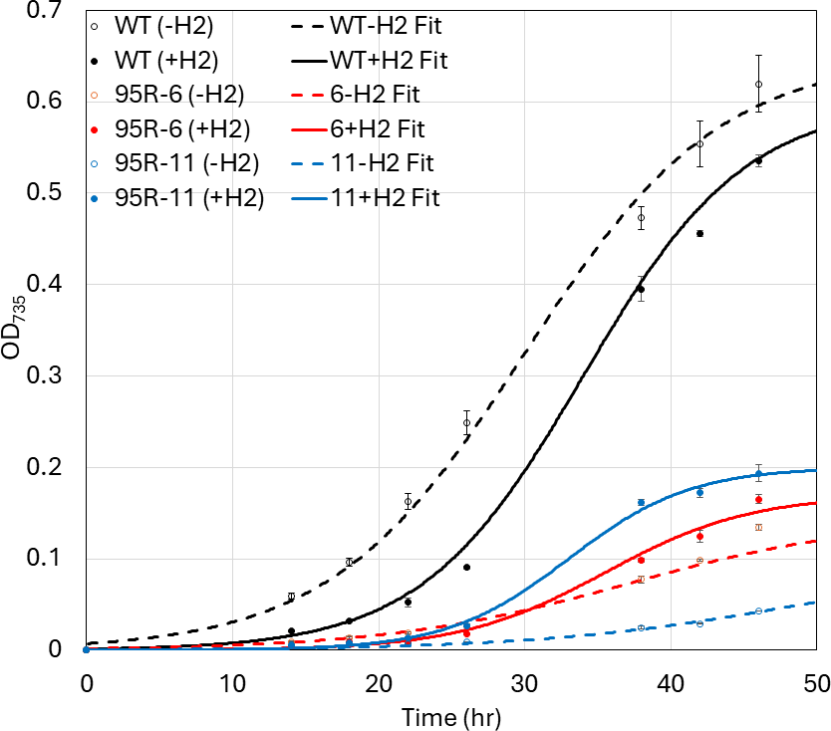
Effect of H_2_ on growth of WT (black) and two different ΔHM1_2993 strains (95R-6, red; 95R-11, blue). Strains were grown in ABYE/4 media in sealed vials. Media were bubbled with either N_2_/CO_2_ (-H_2_, open symbols, dashed lines), or N_2_/CO_2_/H_2_ (+H_2_, closed symbols, solid lines) prior to inoculation. Samples were removed from vials for analysis using syringes so as not to compromise the integrity of the headspace atmosphere. Data points represent the average of technical triplicates, and error bars represent standard deviation of the replicates (*n* = 3).

We compared growth on acetate + bicarbonate after bubbling the medium in sealed vials with N_2_ (80%)/CO_2_ (20%) or N_2_ (75%)/CO_2_ (20%)/H_2_ (5%). The presence of H_2_ in the headspace increased the growth of the ΔHM1_2993 mutants by a variable amount but did not restore it to the level of WT. In contrast, growth of WT cultures was slightly inhibited by the presence of H_2_, for reasons we do not yet appreciate. This suggests that H_2_ can be used as an electron source by the ΔHM1_2993 mutants at some level (Fig. 7).

**Fig 8:**
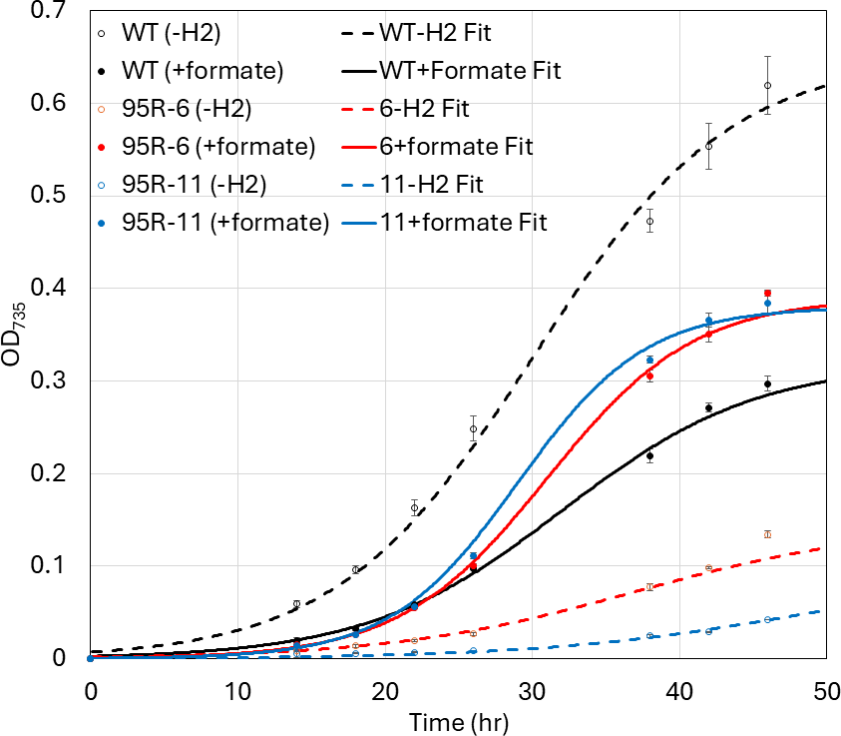
Growth of WT (black) and ΔHM1_2993 (95R-6, red; 95R-11, blue) in ABYE/4 medium in sealed vials sparged with N_2_/CO_2_ and provided with (closed symbols, lines) or without (open symbols, dashed lines) 30 mM sodium formate. Media samples were removed for analysis using needles and syringes, as described in the legend to Fig. 7. Data points represent the average of technical triplicate, and error bars represent standard deviation of the replicates (*n* = 3).

The *H. modesticaldum* genome predicts the presence of a soluble formate dehydrogenase enzyme that oxidizes formate and reduces NAD(P)^+^ (overall reaction: HCO_2_^-^ + NAD(P)^+^→ CO_2_ + NAD(P)H) (2). Thus, heliobacterial cells should be capable of utilizing formate as an electron source, assuming that they can take up formate. We observed a remarkable increase in growth of the ΔHM1_2993 mutants on acetate/bicarbonate upon the addition of 30 mM sodium formate (Fig. 8). The growth rates of the mutants also increased to similar levels as that of WT (from 0.12 h^−1^ and 0.11 h^−1^ to 0.2 h^−1^ and 0.23 h^−1^ for 95R-6 and 95R-11, respectively, WT is typically around 0.2 h^−1^). For unknown reasons, WT grows to lower densities in the presence of formate. Thus, the ΔHM1_2993 mutants grew somewhat better than WT in acetate/bicarbonate/formate medium.

**Fig 9:**
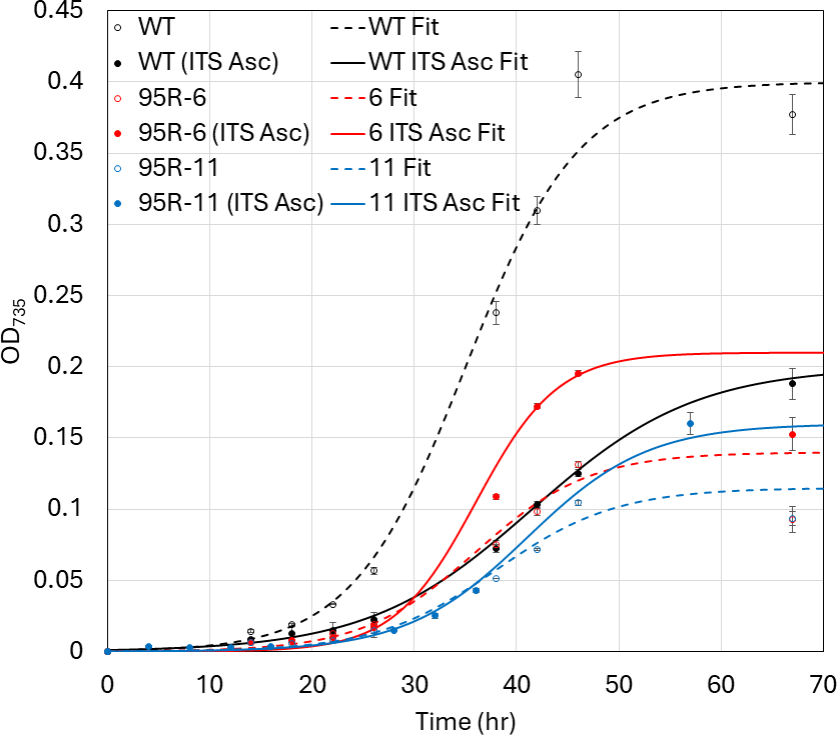
Growth of WT (black) and ΔHM1_2993 (95R-6, red; 95R-11, blue) in ABYE/4 medium in sealed vials sparged with N_2_/CO_2_ and provided with (closed symbols, solid lines) or without (open symbols, dashed lines) 20 mM sodium ascorbate and 10 µM ITS. Media samples were removed for analysis using needles and syringes, as described in the legend to Fig. 7. Data points represent the average of technical triplicate, and error bars represent standard deviation of the replicates (*n* = 3).

Finally, we tested the ability of heliobacterial cells to use ascorbate as an electron donor. Although this molecule has been utilized in several *in vitro* studies of the HbRC from this species (34, 39, 40), it has not yet been tested *in vivo*. We know that ascorbate can reduce the HbRC and cyt *c*_553_ (32, 41, 42) from this species, but it is slow (seconds time scale). Thus, we included the mediator indigo trisulfonate (ITS) at low concentration (10 µM) to mediate electron transfer from ascorbate to cyt *c*, which is responsible for re-reducing the reaction center (Fig. 2). We have shown that ascorbate cannot be used as a carbon source by these cells (see Fig. S7); hence, any effect on growth should be a result only of electron donation. The addition of ascorbate/ITS moderately increased the growth of the ΔHM1_2993 mutants, albeit in a variable fashion, not unlike the addition of H_2_ (Fig. 7). Surprisingly, the addition of the ascorbate greatly reduced the growth of the WT strain. Thus, in the presence of ITS/ascorbate, the mutants grew on acetate/bicarbonate like the WT strain (or slightly better) (Fig. 9).

To explore possible synergistic effects of H_2_ and formate, two electron donors that a soil bacterium is likely to encounter in anaerobic niches, we grew cells on acetate/bicarbonate in 96-well plates open to the atmosphere of the glovebox, and thus to H_2_, and explored the effect of added formate. In the presence of H_2_ alone, WT far outperformed the ΔHM1_2993 mutants. After the addition of formate, however, the growth of WT was slightly suppressed but that of the mutants was greatly increased (Fig. S8). Thus, the mutants all grew somewhat better than WT in this condition.

### Requirement for glutamine in minimal salts medium

**Fig 10:**
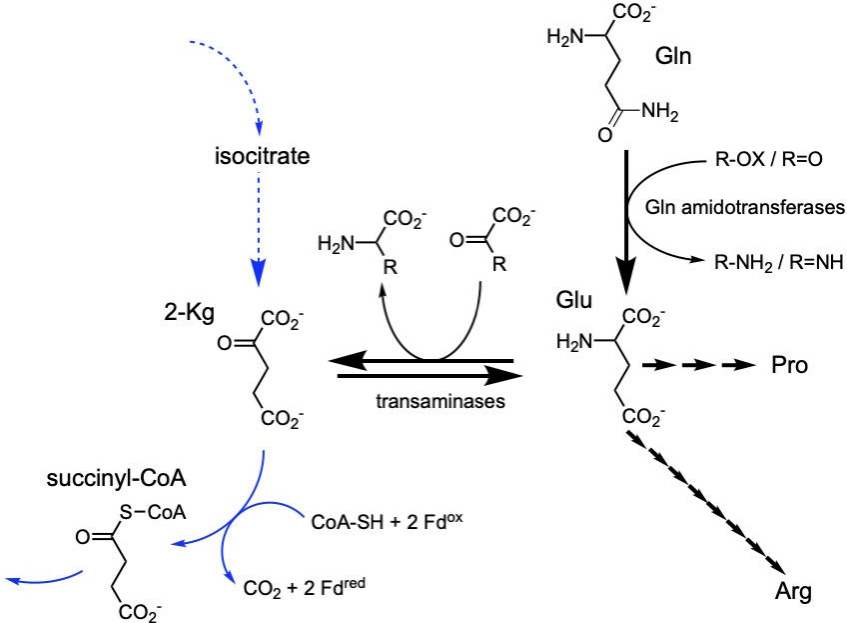
Schematic displaying how glutamine could be incorporated as both a supplemental carbon source for amino acid biosynthesis and an electron source. Use of glutamine as a nitrogen donor by Gln amidotransferases results in the production of glutamate, from which Pro or Arg can be synthesized in three or eight steps, respectively. Use of glutamate as a nitrogen donor by transaminases results in the generation of 2-KG, which can be converted to oxaloacetate by the oTCA cycle (blue arrows, only the next two steps shown), generating six reducing equivalents.

We had severe difficulties getting the ΔHM1_2993 mutants to grow in medium lacking yeast extract. We reasoned that there may be metabolites required by the mutants that they could not synthesize sufficiently to sustain growth. Indeed, the growth of *si*-CS mutants in several organisms necessitated the addition of a downstream TCA cycle product, such as glutamate or arabinose (in the case of *Rhizobium*, which can convert arabinose into 2-KG) (25–27, 43). As discussed in the Introduction section, in heliobacteria, the majority of the flux to 2-KG is through the oTCA pathway, with very little flux in the rTCA direction (7, 44). Thus, we would predict that the lack of the oTCA cycle in the ΔHM1_2993 mutants would result in a deficiency in 2-KG synthesis, which would lead to a lack of the essential amino acids whose synthesis proceeds from this metabolite (i.e., Glu, Gln, Pro, and Arg). Although *H. modesticaldum* appears to take up very few amino acids, it does take up glutamine, which it can use as a nitrogen source (9). Use of glutamine as a donor of ammonia via a glutamine amidotransferase-type enzyme (e.g., carbamoyl-phosphate synthetase, cytidylate synthetase, glutamine-PRPP synthetase) during nucleotide biosynthesis would result in the production of glutamate. This could serve as the starting point for the synthesis of Arg and Pro. Glu would also be converted to 2-KG via a transaminase during biosynthesis of most amino acids (Fig. 10). Entry of 2-KG into the oTCA cycle would result in the generation of six reducing equivalents during its conversion to oxaloacetate (Fig. 1). Thus, we reasoned that if the addition of glutamine allowed sufficient biosynthesis of the other three amino acids, as well as provide reducing equivalents for metabolism, we should be able to grow the mutants in acetate without yeast extract. In fact, this was the case. The ΔHM1_2993 mutants grew in defined minimal salts (MS) medium (4, 38) on acetate/bicarbonate if 10 mM glutamine was included (Fig. S9).

**Fig 11:**
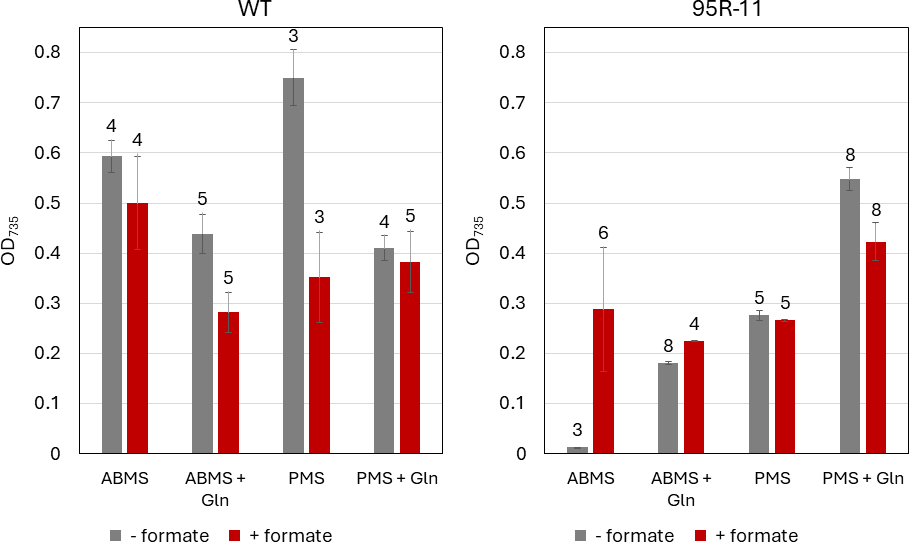
Growth of WT (left) and ΔHM1_2993 strain 95R-11 previously weaned from glutamine (right) in minimal media with pyruvate (PMS) or acetate + bicarbonate (ABMS) as a carbon source, and lacking or containing 10 mM glutamine, and either with (red bars) or without (gray bars) 30 mM formate in the media. Cultures were grown in sealed vials, and portions were removed daily to read with the Epoch spectrometer to obtain OD_735_ values. Each bar represents the maximal OD_735_ achieved by that strain in the specific medium; the number of days required to reach that OD is displayed above the bar. Error bars describe the standard deviation of technical triplicate (*n* = 3).

After adjusting the ΔHM1_2993 mutants to this medium, we reduced the quantity of added glutamine in large steps (from 10 mM to 5 mM, and then to 0 mM) over the course of several months. This progressive reduction of the amount of added glutamine eventually resulted in entrainment of the cells to grow without any added glutamine. We interpret this to mean that the cells were able to increase the flux to 2-KG via the rTCA pathway. In the cells that had been “weaned” from glutamine and grown in a media in which the only organic components were acetate and formate, we could finally see the absolute dependence upon the electron donor - leaving out formate resulted in no growth at all (Fig. 11). Interestingly, addition of glutamine to the “weaned” strain greatly increased growth in the absence of formate. We interpret this to mean that they were able to use the excess glutamine not required for Pro and Arg biosynthesis as an electron source (see Discussion). Addition of formate to the PMS medium did not increase the growth of the ΔHM1_2993 mutant, and in fact, it slightly inhibited the very strong growth in PMS + Gln medium. In all media, the addition of formate to the WT culture either had no effect or inhibited growth.

These strains reached their maximal OD after several days of growth. Although WT strains in all media typically reach their maximal OD_735_ by day 4, most of the knockout strains did not reach their maximal OD_735_ until day 5 or later, except when grown in formate- and glutamine-supplemented acetate media, in which the maximal OD_735_ was attained by day 4.

## DISCUSSION

### Identification of HM1_2993 as the heliobacterial citrate synthase

The data presented in this paper provide sufficient evidence to support the conclusion that gene HM1_2993 encodes a *Re*-citrate synthase, as proposed by Tang et al. (7).

Protein alignments of HM1_2993 with the known and isolated *Re*-CS from *Clostridium kluyveri* and *Syntrophus aciditrophicus* yielded a relatively low identity at around 28%. However, when compared with other known and proposed *Re*-CS genes from other bacterial species, *Dehalococcoides mccartyi, Clostridioides difficile*, and *Thermoanaerobacter sp*. X514, the percent identity increases to 36%–68%. This was discussed by Feng et al. (14) and Marco-Urrea et al. (19); they also proposed that the *Re*-CS from *Thermoanaerobacter* X514 and *Dehalococcoides* CBDB1 may constitute a distinct clade of *Re*-CS from the ones expressed in the majority of clostridia. Our result supports that idea, since the two *Re*-CS genes from *H. modesticaldum* and *Thermoanaerobacter* yield high identity to each other (and a relatively lower identity with the *Dehalococcoides*), but not to the *Re*-CS from the clostridia in which the gene was originally isolated.

Considering the fact that *Re*-CS from other species have often been misannotated as homocitrate synthase or isopropylmalate synthase, there are three loci in addition to HM1_2993 that could possibly encode for an *Re*-CS: *nifV* in the *nif* operon (annotated as homocitrate synthase), as well as *leuA* and HM1_1519 of the *leu* operon (annotated as isopropylmalate synthase and citramalate synthase, respectively). Since these genes are part of well-defined operons, they are very likely to encode the enzyme predicted in the annotation rather than a *Re*-CS, and the protein alignments agree with that assessment. HM1_2993 boasts the highest percent identity of the three possible *H. modesticaldum* genes with the known and putative *Re*-CS sequences from other organisms.

It has previously been shown that deletion of citrate synthase can impact acetate utilization and metabolism, resulting in decreased growth in acetate-based media (45), or in an increase in acetate accumulation due to acetyl-CoA no longer draining into the oTCA cycle (46). The impact of the ΔHM1_2993 mutation on acetate growth further supports the hypothesis that HM1_2993 is the gene encoding *Re*-citrate synthase in *H. modesticaldum*.

When carrying out BLAST searches with HM1_2993, the top eight hits are the other heliobacterial species with sequenced genomes (with conserved identity ranging from 82% to 99%). The fact that this gene is conserved in all members of the Heliobacteriaceae family tested so far is consistent with it having an important metabolic role in all heliobacterial species. The 25 next closest matches from identified species are mostly in the class Clostridia (24/25) and highly concentrated in the order Eubacteriales (23/25), where the family Heliobacteriaceae is situated. All of these genes display high conservation (≥72% identity) and sequence coverage (≥97%), indicating that they likely share the same function; however, all are annotated as “homocitrate synthase.” (The search also pulled out a gene from a member of the phylum Armatimonadota, but this may represent horizontal gene transfer, as a search using that gene as a query identified no other genes from that phylum.) Thus, we conclude that the *Re*-CS gene in heliobacteria is a Clostridial/Eubacterial gene inherited from its non-phototrophic ancestor, and it has been conserved after this lineage became phototrophic due to its key role in central carbon metabolism.

### How heliobacteria use the TCA cycle under different growth modes

Although it may be surprising to some that the key enzyme of such a central metabolic pathway could be non-essential, it speaks to the function of the TCA cycle in this organism. Heliobacteria lack any terminal oxidases and do not appear to be able to use any terminal electron acceptors (e.g., nitrate, sulfate); hence, they do not generate ATP using electron transport to such molecules. Indeed, the fact that the cytochrome *bc* complex (Complex III) is non-essential in this organism, but is required for phototrophy, argues strongly that the mobile cytochrome *c* in heliobacteria is used to reduce the RC and nothing else (13). Instead, as in many anaerobes, the TCA cycle is primarily used for carbon metabolism and to provide precursors for amino acid biosynthesis. The data shown here support the proposal made by Tang et al. (7) that in heliobacteria grown on pyruvate the TCA cycle is split, with the oTCA cycle from citrate to 2-KG used to provide the starting points for the biosynthesis of the Glu/Gln/Pro/Arg family of amino acids. OAA can be made from PEP by PEPCK when needed for biosynthesis of the Asp/Asn/Thr/Met family of amino acids and pyrimidines, whereas excess OAA can be converted to PEP for carbohydrate synthesis. There is also a flux in the rTCA pathway from OAA to succinyl-CoA, but a very low flux to 2-KG, presumably due to low KFOR activity in that growth mode (7). It is, however, difficult to explain this flux through the rTCA cycle, since none of the intermediates after OAA (i.e., malate, fumarate, succinate, succinyl-CoA) are known metabolites required for the biosynthesis of any amino acid or nucleotide. Recent work in our group is consistent with a model in which the rTCA cycle diverts from succinyl-CoA and proceeds back to OAA, forming another cycle involving methylmalonyl-CoA as an intermediate and producing propanoate as a fermentation product (Gisriel et al., manuscript in preparation). This cycle would also be able to process the fumarate produced as a byproduct during biosynthetic steps in which aspartate is used as an amino group donor (e.g., argininosuccinate lyase in arginine biosynthesis or adenylosuccinate lyase in purine biosynthesis).

In light of this model, one can understand why the loss of CS has little impact upon heliobacterial growth on pyruvate, since pyruvate can be used as an electron source independent of the oTCA cycle. Heliobacterial cells have no use for citrate or isocitrate. As long as they have an electron donor, the only difficulty the ΔHM1_2993 mutant cells have is in producing sufficient 2-KG to support the synthesis of the Glu family of amino acids, which can be alleviated by the addition of glutamine or yeast extract. Weaning of the mutant cells from glutamine likely involves the upregulation of KFOR to allow sufficient flux to 2-KG by the rTCA pathway. It is impressive that they are able to re-route carbon flow so effectively within a few dozen generations. We suggest that selective pressure has resulted in mutations altering the expression of enzymes that may be a bottleneck, chief among them being KFOR, since it seems to be limiting in pyruvate-grown cells (7).

In contrast to growth on pyruvate, we propose that WT heliobacteria growing on acetate operate the TCA cycle fully in oTCA mode, for the primary purpose of oxidizing acetate completely to CO_2_ and harvesting eight reducing equivalents to drive reductive anabolic reactions (especially PFOR). There is no other way they have of performing conversion of acetate to CO_2_. Thus, for every four acetates converted to pyruvate by reductive carboxylation at PFOR, one acetate would have to be sacrificed to provide the electrons. There are, of course, numerous reductive reactions used in other anabolic pathways. In addition, the cycle would also provide 2-KG for amino acid synthesis. A prediction of this model is that the flux through the oTCA pathway in heliobacteria would be significantly higher when grown on acetate than on pyruvate.

This model explains why no electron donor (besides acetate itself) was required for the growth of WT cells on acetate. It also explains why the CS deletion mutants cannot grow in this mode unless provided with an electron donor. Loss of CS would completely block the entry of acetate (as acetyl-CoA) into the oTCA cycle. It is interesting to note that none of the electron donors tested here (H_2_, formate, ascorbate) interact with the TCA cycle, and none of the oxidation products (protons, CO_2_, dehydroascorbate) are organic molecules that can be utilized by these cells. Moreover, the addition of these electron donors usually results in an inhibition of WT growth. The fact that all three donors allow the growth of the CS mutants on acetate (at some level) argues strongly that all they are providing is electrons.

One can then ask what the TCA cycle is doing in the CS deletion mutants growing on acetate in the presence of formate, which seems like the best growth mode. We propose that OAA is made via PEPCK as usual and that some flux proceeds from OAA down the rTCA pathway as in pyruvate-grown cells, but likely to make 2-KG rather than propanoate (as they are electron deficient). It takes some entrainment to get these mutant cells to the point where the addition of glutamine is no longer necessary, and their growth is still slower than WT, but there is no other way for them to make the Glu family of amino acids in the absence of Gln. However, this pathway can clearly be operated in the opposite direction (from 2 KG to OAA) in some cases, as glutamine can replace the electron donor at least partially (Fig. 11). We propose that the use of glutamine and glutamate as nitrogen donors in nucleotide and amino acid biosynthesis would result in the production of some 2-KG, which could be converted to OAA, in the process producing six reducing equivalents (Fig. 10).

Analysis of its genome has revealed *H. modesticaldum* to have the potential for altering its carbon metabolism in interesting ways. This study confirms the flexibility of the organism’s carbon metabolism. The deletion of a central enzyme to their carbon metabolism, *Re*-citrate synthase, has furthered this idea, provided clues as to the functions of their existing carbon metabolic pathways, and has expanded the possibilities for alterations that are attainable in this organism.

## SUPPLEMENTAL MATERIAL

Supplemental material aem.01772-24-s0001.pdf Table S1; Figures S1 to S9.

ASM does not own the copyrights to Supplemental Material that may be linked to, or accessed through, an article. The authors have granted ASM a non-exclusive, world-wide license to publish the Supplemental Material files. Please contact the corresponding author directly for reuse.
