# Supplementary material for: Deletion of Re-citrate synthase allows for analysis of contributions of tricarboxylic acid cycle directionality to the growth of Heliomicrobium modesticaldum
Source: Appl Environ Microbiol. 2025 Mar 6;91(4):e01772-24. doi: 10.1128/aem.01772-24 (PMC12016533; doi:10.1128/aem.01772-24)
Supplement: Supplemental material — Table S1; Figures S1 to S9. [file aem.01772-24-s0001.pdf]

Supplemental Materials - Tables

Table S1. Protein sequences of known and putative *Re*-citrate synthases, as well as sequences of other loci potentially coding for *Re*-citrate synthase in *H. modesticaldum*. Sequences were found using NIH, NCBI, and Biocyc databases. Gene info lists the accession number, the enzyme name (if identified), and whether the enzyme is, as of yet, only a putative sequence.

Species	Gene name	Sequence	Source
H. modesticaldum ICE1	HM1_2993	MNNRQITIVDTTLRDGEQTAGVVFANKEKIRIAKMLDEIGV<br>HQIEAGIPVMGGDEKETIAKICKMGLKASIMGWNRAVISD<br>IQHSIDCGCDAVAISISTSDIHIQHKLRTSREWVLESMMKA<br>VDFAKSQGLYVSINAEDASRSDIEFLIQFATEAKKLGANRL<br>RYCDTVGIMEPFTIYEHIKRLREAVDIDIEMHTHDDFGMAT<br>ANAIAGVRAGATHIGVTVNGLGERAGNAALEEVVMALKY<br>CDNIDLGFKSEKFRELCEYVSKASGRFLPTWKSIVGSNMF<br>AHESGIHGDGVLKNPKTYEVISPEEVGLTRQIVIGKHSGTA<br>SIKAKFREFEIDMTEVQAAEVLARVRETAVELKRPLFDKEL<br>MFIYEELYGYPEH	(7, 10)
H. modesticaldum ICE1	NifV<br><br>(Homocitrate Synthase)	MRQRVWLMDTTLRDGEQTPGVAFCPQEKALLARRLAEA<br>GVHEIETGVPAMGEDEQETIARIVKLNLPTRVTTWNRAVIS<br>DLEASLNCGVRSVAICLPSSDQQITQKLRQSRQWVLDQ<br>MGACVRRAKAEGLYVVIGLEDASRADPQFLIQLGLEAERL<br>KVNRLRISDTLGILDPIRTFNLFDRLTSSLSIPLEIHAHNDL<br>GMATANTVSAIQAGAKAASVTVCGLGERAGNAPLEEVAL<br>ALRQCCAADTRINLALLPALCRLVSWSTRRPIPFSKPVVG<br>RDAFTHASSIHVDGLNKDRSNYEAYPPEYVGRRHRIAFGK<br>YSGRKLLAELLKAQGHDLDQEEMTQLLLNVRQLSQVLKR<br>PLRQHDIVDLIFGETSAQRDMRA	(10)
H. modesticaldum ICE1	LeuA<br><br>(2-Isopropylmalate Synthase)	MSKRVVIFDTTLRDGEQSPGVSLNLHEKLEIAQQLARLGV<br>DVIEAGFPIASPGDFEAVKAVAEQVRGPVICALARANRKDI<br>ERAAEALRGAEEARIHTFIATSPIHMQHKLRMEPDKVLDTA<br>VDAVKLAKSFTSNVEFSAEDAFRSDVGFLCRIFSAAIEAGA<br>TTINIPDTVGYATPQEFGAFIKAIINGTPNIDKAIVSVHCHN<br>DLGLAVANTLAALENGALQVEGTINGIGERAGNASLEEVI<br>MALYTRKPFYNLETSINKSEIYRTSRLVSNLTGMLVQPNKA<br>IVGKNAFAHESGIHQDGVLKERTTYEIMNPEMIGIFTNNIV<br>LGKHSGRHAFRERLKELGYSLDDEKLTKAFARFKALADRK<br>REITDEDLVVLVEDELRAFPEAYSLEYLHITSGTVLVPTATV<br>RLRREEENFEEASCGDGPVDAAYKAIEKITGTGARLASYAI<br>SATTAGEDSQGEVSVKLEREGRFYTGRGVDTDIIVASAKA<br>YLNAVNKIVFDGLPKAKTERAV	(10)
H. modesticaldum ICE1	HM1_1519 (Citramalate Synthase)	MDRVFIYDTTLRDGTQGEGISLSVEDKVKIAARLDQLGVAY<br>IEGGWPGSNPKDMEFFQRAQQMTWKHAKIAAFGSTCRP<br>GSEACDDPNLRALIESGAPVVTIFGKSWDFHVTAALRTTL<br>EENLRLVRDSITFLKNQGREVIFDAEHFYDGYKGNPAYAK<br>EVVMTAEKAGADWIVLCDTNGGTLPHDVLSITQEMVFTLR<br>APVGVHVHNDGDLAVANSIMGVMAGARQVQGTMNGYG	(10)

		ERCGNVNLCSVIPNLQLKMKMECLPEGKLQTLTDAAHFV<br>GEIANMPLRNDMPFVGHSAFAHKGGIHVSALMKDPGTY<br>EHIQPEAVGNHRRVLVSELSGMSNVIYKAKELGLDVNRQ<br>NADTKQIIEQIKNLEHQGFQFEGAEASFEVLLRRAFGEDP<br>VPFVLDSIRLIIEKRSDADFTSEAMIKLRVGDQVVHTAAEG<br>NGPVNAVDNALRKALLSHYPFLAECHLTDYKVRVLDGKD<br>ATEAKVRVLIETRDSHDAWGTVGVSTNIIEASWQALMDSF<br>LYGYMRERARASIESSRPA	
Syntrophus aciditrophicus	SYN_02536, CP000252.1<br>(*Re*-Citrate Synthase)	MAKWNPQKRVLNHEHTRFWRFELRDVDEPNLQKEVFPY<br>DEVSRIDFDHRIIPIQPAEEIFITDTTFRDGQQARPPYTTQQ<br>IVDLYQMMSRLGGYNGIIRQTEFFLYSNRDKEAVRMCQD<br>LGLQYPEITGWIRAAREDIPLVKEAGLKETGILTSVSDYHIF<br>LKLNMTRSQALEEYLGIVKAILDAGIVPRCHFEDITRADIYG<br>FCIPFAIELMKLREESGVDIKIRLCDTMGYGVTYPGASLPR<br>GVDKLVRAFIDDADVPGRLLEWHGHNDFHKALINATTA<br>WLYGCSAANSTLLGLGERTGNPPIEGLIIEYIGLMGKTNGI<br>DTTVITDIANYFKNEIEYKIPSNYPFVGADFNVTRAGVHAD<br>GLIKSEEIYNIFNTTKILKRPIVPMITDKSGKAGIAYWINSHF<br>GLSGDSTVDKRHPGISKINKWIADEYELGRVTTISTEELEA<br>KVRKYMPELFMSDLERIKFKAAEAAIAVLRKIIDDPAMKTM<br>QPELQEPVMQRFIEEYPSIQFAYVVDMNGKKTTRNITNIV<br>DRAKYENYGVGTDQSDREWFIKPLQTGKLHVTDFYISKM<br>TGALCFTVSEPITDDNDDMVGIFGVDIRVEDLVKEPEYIAE<br>ATQIALKAEYDAKYKSDHWL	(21)
Clostridium kluyveri DSM 555	CKL_0973<br>(*Re*-Citrate Synthase)	MKKCSYDYKLNNVNDPNFYKDIFPYEEVPKIVFNNIQLPM<br>DLPDNIYITDTTFRDGQQSMPPYTSREIVRIFDYLHELDNN<br>SGIIKQTEFFLYTKKDRKAAEVCMERGYEFPEVTSWIRADK<br>EDLKLVKDMGIKETGMLMSCSDYHIFKKLKMTRKETMDM<br>YLDLAREALNNGIRPRCHLEDITRADFYGFVVPFVNELMK<br>MSKEANIPIKIRACDTLGLGVPYNGVEIPRSVQGIIHGLRNI<br>CEVPSESIEWHGHNDFYGVVTNSSTAWLYGASSINTSFL<br>GIGERTGNCPLEAMIFEYAQIKGNTKNMKLHVITELAQYFE<br>KEIKYSVPVRTPFVGTDFNVTRAGIHADGILKDEEIYNIFDT<br>DKILGRPVVVAVSQYSGRAGIAAWVNTYYRLKDEDKVNK<br>NDSRIDQIKMWVDEQYRAGRTSVIGNNELELLVSKVMPE<br>VIEKTEERAS	(24)
Dehalococcoides mccartyi sp. CBDB1	CAI83711	MGKIFIIDVTNRDGVQTARLGLSKLEKTLINIYLDEMGIFQS<br>EFGFPTTKHERGYVEANLELAKMGVIKNLRLEGWIRAIVA<br>DVDLAFRRAPSLKHLNLSISTSEQMINGKFQGRKVFKDIIE<br>DMTIAVNAAYAKGAETVGVNAEDASRTSIVNLIEFGKAAKE<br>VGATRLRYCDTLGYDNPFTIYETARTLAEKVGMPIEIHCHG<br>DLGMAIGNSLAGAKGVIDGGQDVYVNTTVNGIGERAGN<br>ADLVAFLLAILKSKGFGEKYQLGHEVDLSKAWKIARFASY<br>AFDVEIPINQPGVGRNCFAHASGIHADGVIKDSQNYELY<br>GYEELGRGEALMVETGREICAGQYSGISGFRHVMGNMS<br>VELPEDKDEANKILELVRYANVEAHKPLVEDELIFIAKYPEI<br>SRRLLTLTPLMND	(19)
Clostridium difficile	AksA	MCVISKDRAKEIKIVDTTLRDGEQTAGVVFANREKIMIAEM<br>LSDLGVDQIEVGIPTMGGDEKNVIKHICSRNLKSDIMAW<br>NRAVIKDVEESISCGVDAVAISISVSDIHIENKLRTSRGWVL	(24)

	(Re-citrate synthase (trans-homoaconitate synthase))	ENMAKTVEFAKKNGLYVSVNGEDASRADIDFLTEFINVGK<br>QAGADRFRYCDTVGVMNPFSIKNAIETLYERTNFDIEMHT<br>HNDFGMATANALAGIAAGANYVGVTVNGLGERAGNAAL<br>EEVLMALKCVYKCDLNNIDTRKFRGICEYVAQASGRILPT<br>WKPVVGDNMFIHESGIHADGALKDPHNYEPFDPSEVNL<br>ERKIVIGKHSGRAAVVNKLSEYEMYISPENATKLLNAIRATS<br>IRLKRSLMDKEILQLYCDILAHEKGTTEEEAVRGSYI	
Clostridium acetobutylicum ATCC 824	CAC0970<br>(putative)	MKELNLKDVEEPNLYRDIFPYHEVPKIKFSTDEIKVDIPDEI<br>WITDTTFRDGQQSMTPFTVEQIVTIFDYLNKLDNNTGVIR<br>QTEFFLYTNRDKEALMECMNRGYKFPQITTWIRANKDDF<br>KLVKDIGIKETGILMSCSDYHIFKKLKMTRTETYNKYVEIVE<br>EALSNGIVPRCHLEDITRADFFGFVVPLVNKLMELSNKYGI<br>QVKIRACDTLGLGVAFPGVELPRSVPAIISGLRKYCGVPST<br>ALEWHGHNDFYYVVPNATAAWLHGCSAVNTTLLGIGER<br>TGNCPLEGMVFQYCQLKGNPGMNLHAITEMSKYFENSM<br>KYEIPPRTPFVGTDFNVTRAGIHADGILKDQEIYNIFDTEKIL<br>DRPVLVAVNEYSGLAGIAAWINTYFKLNKENEVDKKDSRV<br>AEIKKWVDNLYENGRTTPITNKELEIEAKMYFKELIDVENTR<br>AS	(28)
Clostridium thermocellum	CL01313_RS03665<br>(putative)	MIEFNKKTNTLEQVQYKYTLQDVSEPNLYRDIFSYDEIPKC<br>TFNHRKVPMAPPDEIWITDTTFRDGQQSRAPYTVEQIVHL<br>YDLLHKLGGPKGIIRQCEFFLYSDRDKQAVYKCLERGYKY<br>PEVTSWIRATKSDFQLAKDMGMKESGILVSCSDYHIFKKL<br>NMTRKQALEHYMSIVKSAIEVGIRPRCHFEDITRADFYGFV<br>VPFAIELRKLMEESGVPIKIRACDTLGYGVSYPGAALPRSV<br>PGIIYGLRHYAGFPSELIEWHGHNDFYKAVCNAATAWLY<br>GASAVNCSLLGIGERTGNTPLEAMVIEYAQLRGTTDSMDT<br>TVITEIAEYYEKELGYQIPPRTPFVGKHFNVTQAGIHADGLL<br>KDEEIYNIFDTAKLLNRPVGVAINQTSGLAGIAHWINSHFG<br>LEGAKRIDKRDERVVKIKEWVDEQYKAGRVTSIGDDELEE<br>VIRKLAPEIFDLAL	(18)
Thermoanaerobacter X514	Teth514_0415<br>(putative)	MTLKKGKKVYIVDTTLRDGEQTAGVVFANNEKIRIAQMLD<br>EIGIDQLEVGIPTMGGDEKETVTKIAKLGLNASIMAWNRA<br>VVKDVQESLECGVDAVAISVSTSDIHIEHKLKKTRQWVLD<br>NMTEAVKFAKKEGVYVSVNAEDASRTDMNFLIEFAKCAK<br>QAGADRLRFCDTVGFLDPFKTYDMVKAIKEAVDIDIEMHT<br>HNDFGMATANALAGMRAGANFIGVTVNGLGERAGNAAL<br>EEVVMALKHVYKIDLGIDTTRFREISEYVALASGRQLPAWK<br>AIVGTNVFAHESGIHVDGALKNPHTYEIFNPDEVGLERQI<br>VIGKHSGTAALINKFKEYGRVLTEEEANLLLPHVRKLAIQLK<br>RPLFDKELMYLYEDVIKNREKAI	(14)

Supplemental Materials - Figures

	HM1_2993 (*H.modesticaldum*)	Terth514_0415 (*Thermoanaerobacter*)	AksA (*C.difficile*)	NifV (*H.modesticaldum*)	LeuA (*H.modesticaldum*)	CA183711 (*D.mccartyi*)	HM1_1519 (*H.modesticaldum*)	Syn_02536 (*S.aciditrophicus*)	CLO1313_RS03664 (*C.thermocellum*)	CAC0970 (*C.acetobutylicum*)	Re-CS (*C.kluyveri*)
HM1_2993 (*H.modesticaldum*)	100.00	68.56	61.93	46.22	38.14	36.23	30.41	28.03	29.48	27.34	27.17
Terth514_0415 (*Thermoanaerobacter*	68.56	100.00	65.42	44.66	40.99	36.04	28.53	30.31	29.77	27.40	27.70
AksA (*C.difficile*)	61.93	65.42	100.00	43.44	37.24	35.70	28.41	30.06	29.18	28.85	27.62
NifV (*H.modesticaldum*)	46.22	44.66	43.44	100.00	35.17	30.05	27.47	25.70	25.94	27.54	24.32
LeuA (*H.modesticaldum*)	38.14	40.99	37.24	35.17	100.00	30.03	28.33	26.43	26.91	28.01	23.10
CA183711 (*D.mccartyi*)	36.23	36.04	35.70	30.05	30.03	100.00	23.60	24.55	26.51	25.54	27.59
HM1_1519 (*H.modesticaldum*)	30.41	28.53	28.41	27.47	28.33	23.60	100.00	23.81	24.87	24.41	26.09
Syn_02536 (*S.aciditrophicus*)	28.03	27.40	30.06	25.70	25.43	24.55	23.81	100.00	60.00	51.95	49.33
CLO1313_RS03664 (*C.thermocellum*	29.48	29.77	29.18	25.94	26.91	26.51	24.87	60.00	100.00	60.59	60.76
CAC0970 (*C.acetobutylicum*)	27.34	30.31	28.85	27.54	28.01	25.54	24.41	51.95	60.59	100.00	65.54
Re-CS (*C.kluyveri*)	27.17	27.70	27.62	24.32	23.10	27.59	26.09	49.33	60.76	65.54	100.00

Figure S1. Percent Identity matrix of known and putative *Re*-citrate synthase protein sequences with the potential *Re*-citrate synthases from the heliobacterial genome. Percent identities were found using the protein-protein BLAST alignment tool from NIH. Sequence identifier or enzyme name is displayed with the host species name in parenthesis. Sequences are listed in **Table S1**.

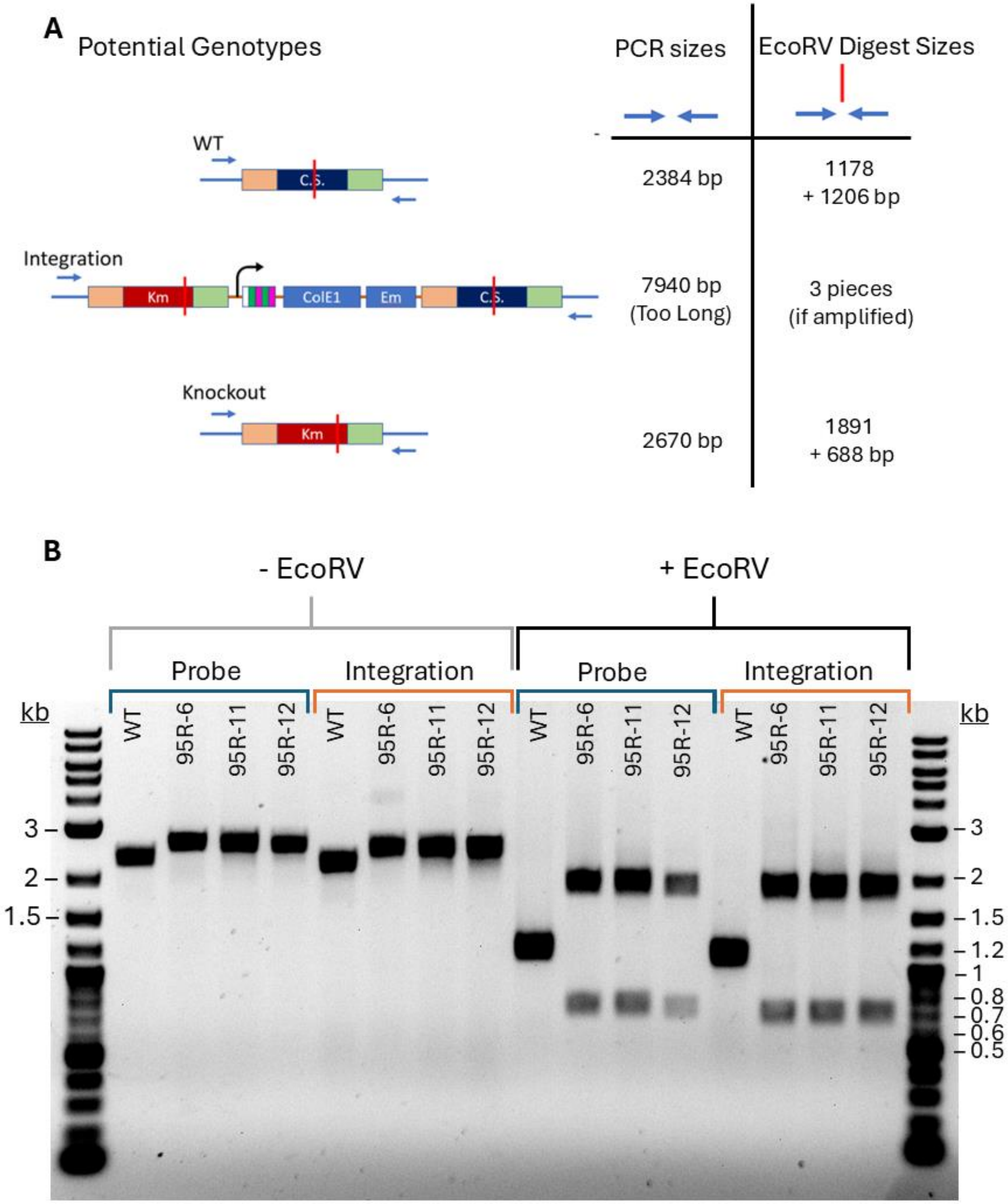

Figure S2. A) Set-up and expectations of PCR/digest of genomic DNA to probe the presence of HM1_2993 (blue) or *aph3* (red, labelled "Km"). Two sets of primers were used,

a “Probe” pair (blue arrows) and an “Integration” pair (orange arrows). Probe primers (blue arrows) anneal to chromosomal DNA but not to any sequences in plasmid pAL95R, while the Integration primers (orange arrows) anneal within the “upstream/downstream” sequences used for homologous recombination. Considering the length of the integrated sequence, “Probe” primers likely would be unable to amplify the entire locus. The “Integration” primers would be able to detect integration of the plasmid. The predicted sizes of the PCR products from HM1_2993 and *aph3* are similar: 2384 and 2670 bp, respectively for the “Probe” reaction, and ~100 bp shorter for the “Integration” reactions. Diagrams of the three potential genotypes, “WT”, “Integration”, and “Knockout” are provided to the left. Locations of EcoRV sites are indicated by red vertical lines. (Note that there are two possible orientations for the “integration” genotype; only one is shown here.) On the right are provided the expected sizes of the PCR products for each genotype and primer setup, and expected sizes of the PCR products when they are subsequently digested with EcoRV. **B)** Agarose gel of the PCR products obtained using genomic DNA from WT and the three putative knockout strains (95R-6, 95R-11, 95R-12) as templates (95R-12 displayed additional growth deficiencies and was not used in subsequent studies). PCR products were generated using either the “probe” or “integration” primers. The right side shows the products after digestion with EcoRV (+EcoRV).

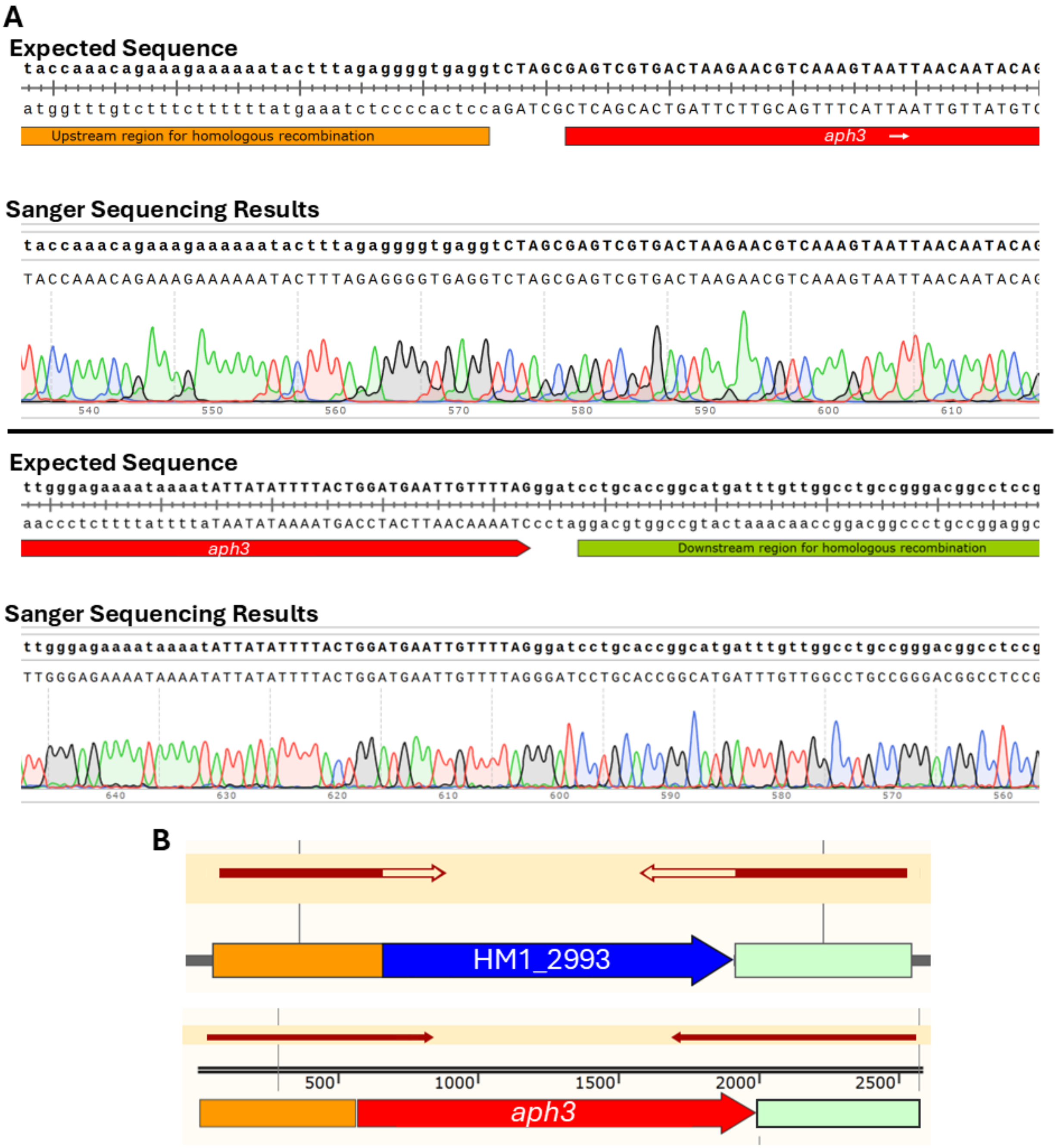

Figure S3. A) Expected (top) sequence of the amplicon from the HM1_2993 locus using genomic DNA from *H. modesticaldum* as template and results from Sanger sequencing (bottom). Panel A focuses on the junctions between the inserted *aph3* gene and the "upstream" and "downstream" flanking regions used to drive homologous recombination.

Chromatograms show raw sequencing data from PCR products of genomic DNA using “Probe”primers. These sequences exactly match the results expected for replacement of HM1_2993 on the chromosome with *aph3*. **B)** Sanger sequencing results of the PCR product using “probe” primers is shown as thin red arrows above the two possible chromosomal versions: original chromosome with HM1_2991 still in locus, and edited chromosome with *aph3* replacement. Solid colored arrows represent sequences that align with the predicted sequences, while open sections represent a lack of alignment.

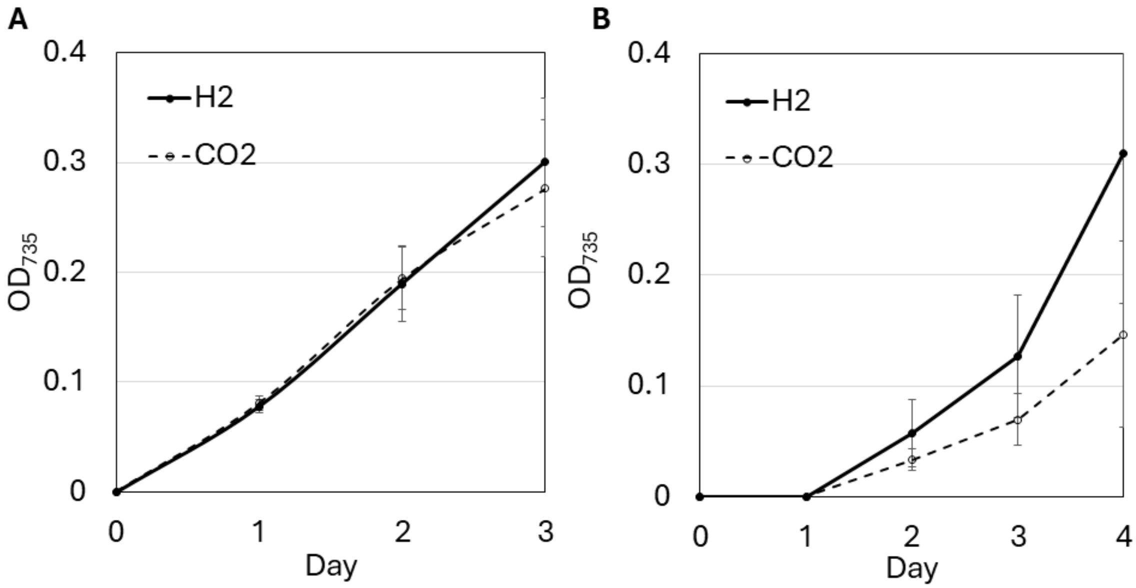

Figure S4. WT *H. modesticaldum* growth with (H2) and without hydrogen (CO2) in acetate bicarbonate minimal media. Displayed is growth of strains upon introduction to ABMS media bubbled with 80% N_2/20% CO_2(1st generation, **A**) and either exposed to the $H_2/CO_2/N_2$ atmosphere of the anaerobic chamber (H_2) or never opened to keep the 80% N_2/20% CO_2 headspace (CO_2), and the growth of strains after adjustment to the media types (2nd generation, **B**). Points represent the average OD_{735} of three separately grown WT strains, and error bars represent the standard deviation (n=3).

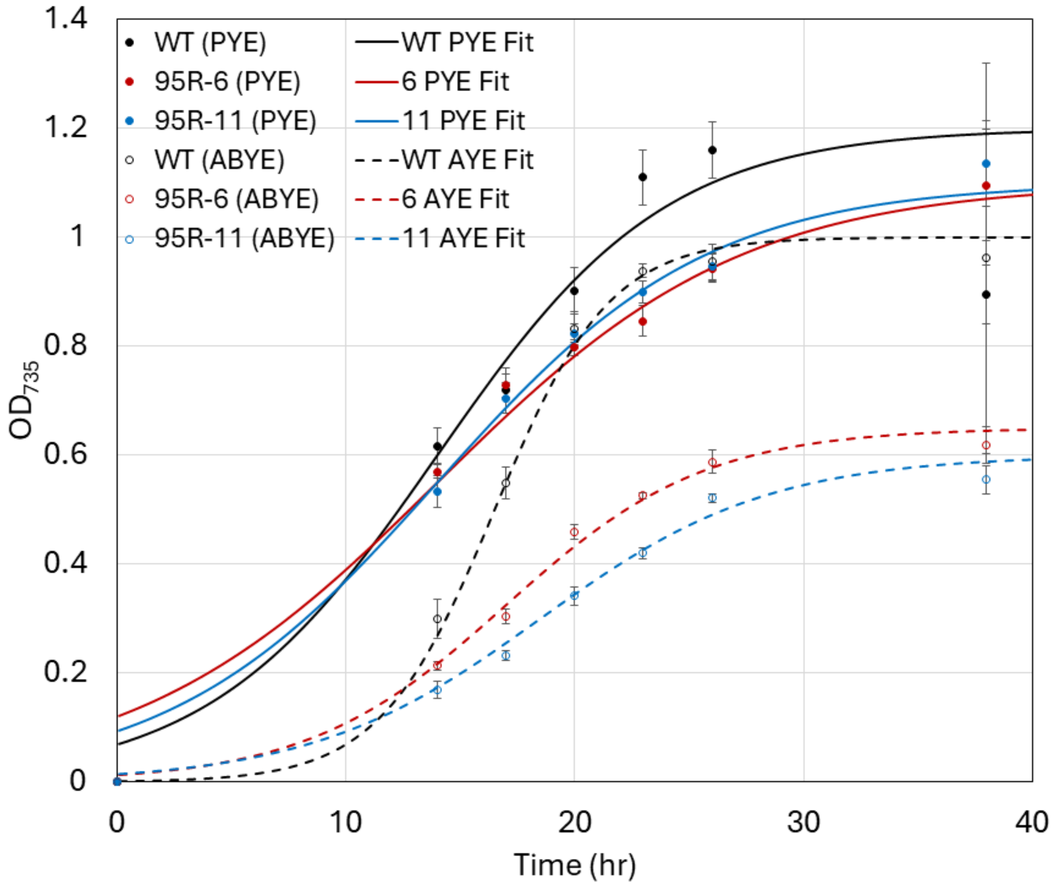

Figure S5. Effect of carbon source in YE-based media on the growth of WT and ΔHM1_2993 strains (95R-6, 95R-11). All strains were grown with pyruvate as a carbon source (PYE, solid circles, solid lines), or with acetate + bicarbonate as a carbon source (ABYE, open circles, dashed lines) in rich media with yeast extract in 96-well plates open to the $H_2/CO_2/N_2$ atmosphere of the anaerobic chamber. Data points represent the average of three separately grown cultures of each strain type. Lines represent fittings of the points to a logistic function. Error bars represent the standard deviation of technical triplicate (n=3).

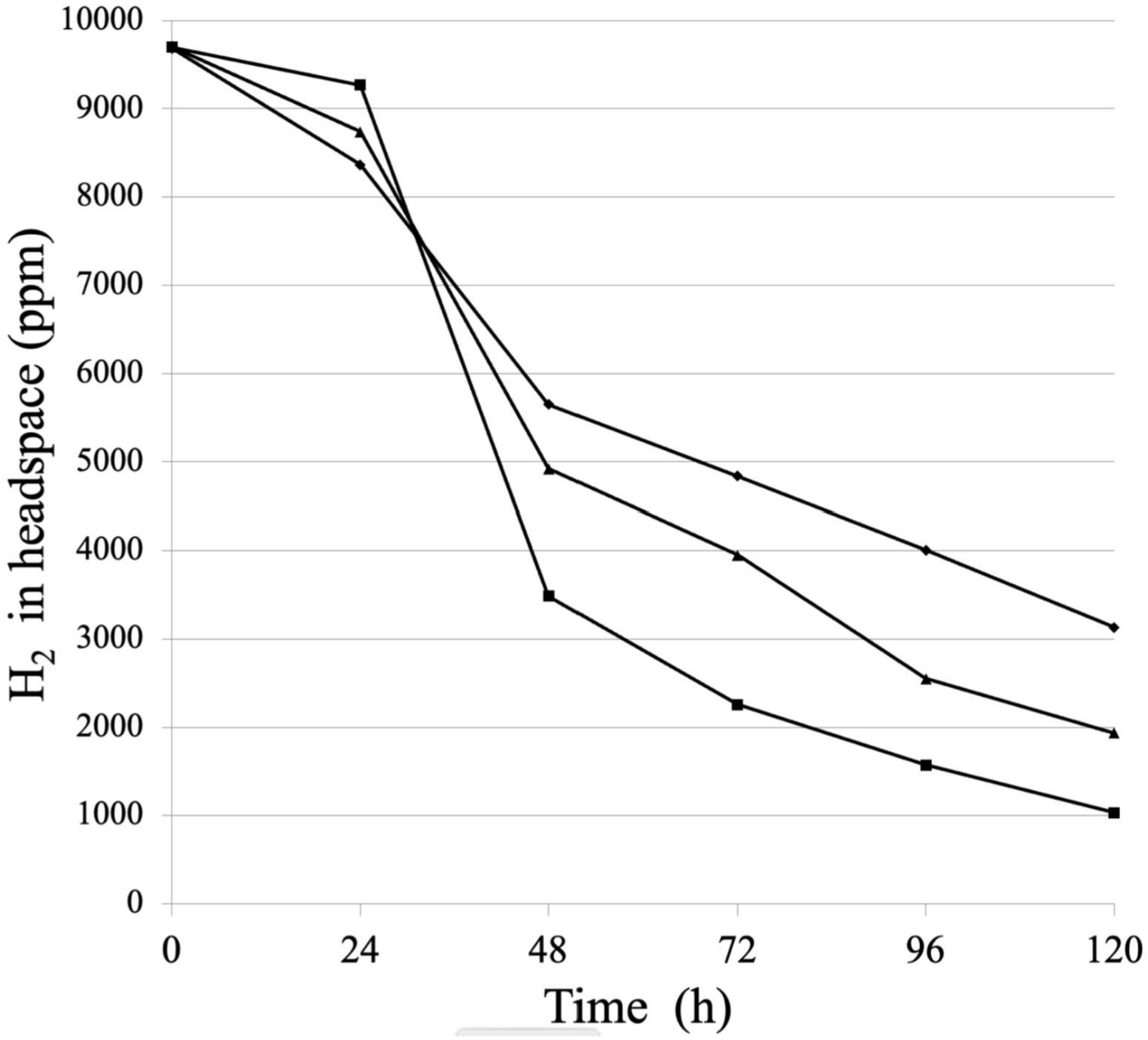

Figure S6. Hydrogen uptake by *H. modesticaldum*. Cultures were grown in pyruvate-based media in sealed bottles with 5% H_2/ 95% N_2 in the headspace (75 mL culture, 20 mL headspace). Headspace samples were monitored once per day by removing 100 µL and injecting it into a gas chromatograph with thermal conductivity detector. Three independent bottles were grown, inoculated from the same starter culture. Light scattering (OD_{620}) was used to follow growth (inset). On average, 2.0 ± doublings occurred in the first day and 1.2 ± 0.06 doublings occurred in the next 4 days.

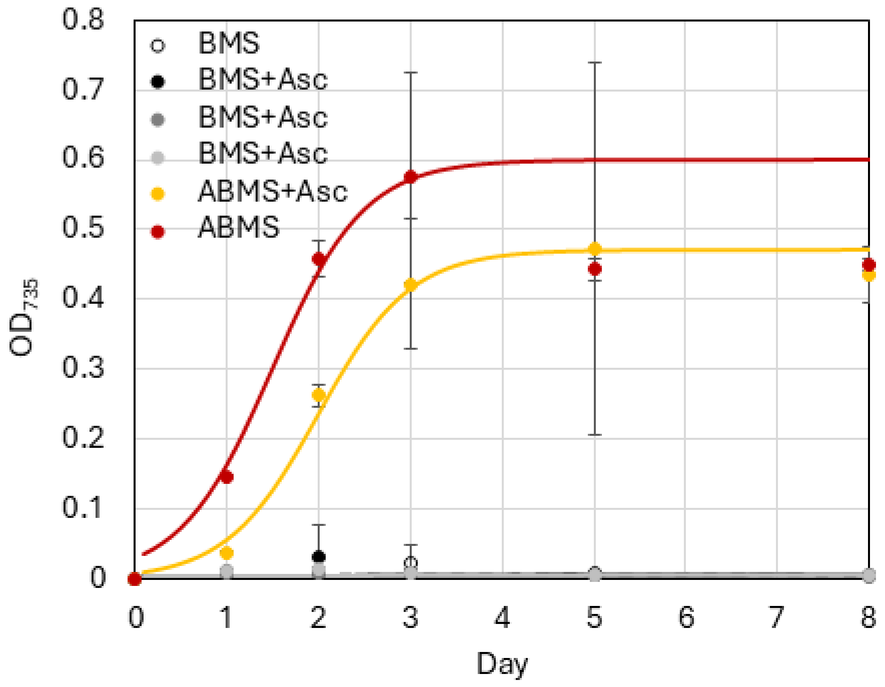

Figure S7. Growth of WT *H. modesticaldum* cells in bicarbonate minimal media (BMS) supplemented with 20 mM ascorbate (+Asc, black and various grey), 20 mM acetate (ABMS, red), or both (ABMS+Asc, yellow). Cultures were grown in sealed vials (headspace containing $H_2/CO_2/N_2$, matching that of the anaerobic chamber), and portions aliquoted to read using the Epoch spectrophotometer. Points represent the average OD_{735} and error bars represent standard deviation of technical triplicate (n=3). Lines represent fittings of points to a logistic equation. Three separate cultures of WT were inoculated into BMS media supplemented with Ascorbate; OD_{735} of all three are displayed separately (black, dark grey, light grey). After day 3, ABMS cultures are fully grown and begin to form sediment in bottom of vials, leading to large error and varying ODs in later days.

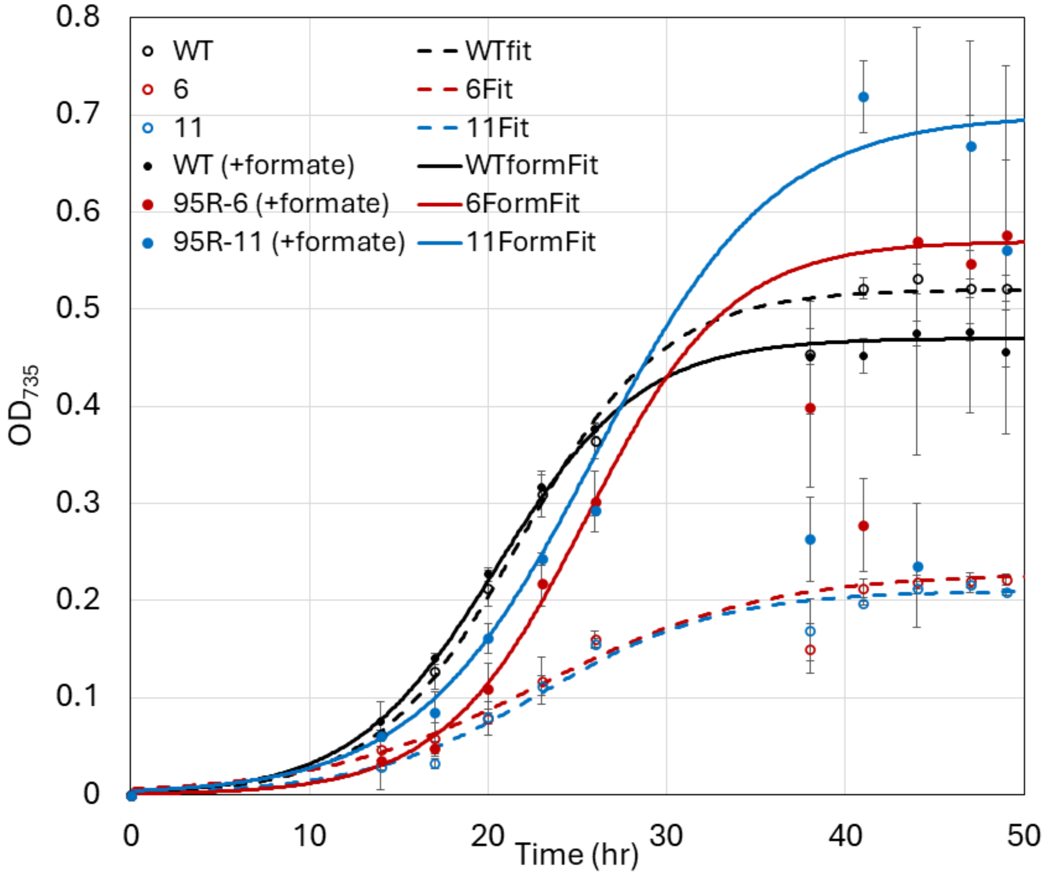

Figure S8. Growth of WT (black) and ΔHM1_2993 (6, red; 11, blue) in ABYE/4 media in open 96-well plates with continual access to the $H_2/N_2/CO_2$ atmosphere and provided with (closed symbols, lines) or without (open symbols, dashed lines) 30 mM sodium formate. Data points represent the average of three separately grown replicates of each strain, and error bars represent the standard deviation of these replicates (n=3). Lines represent the fitting of the points to a logistic equation.

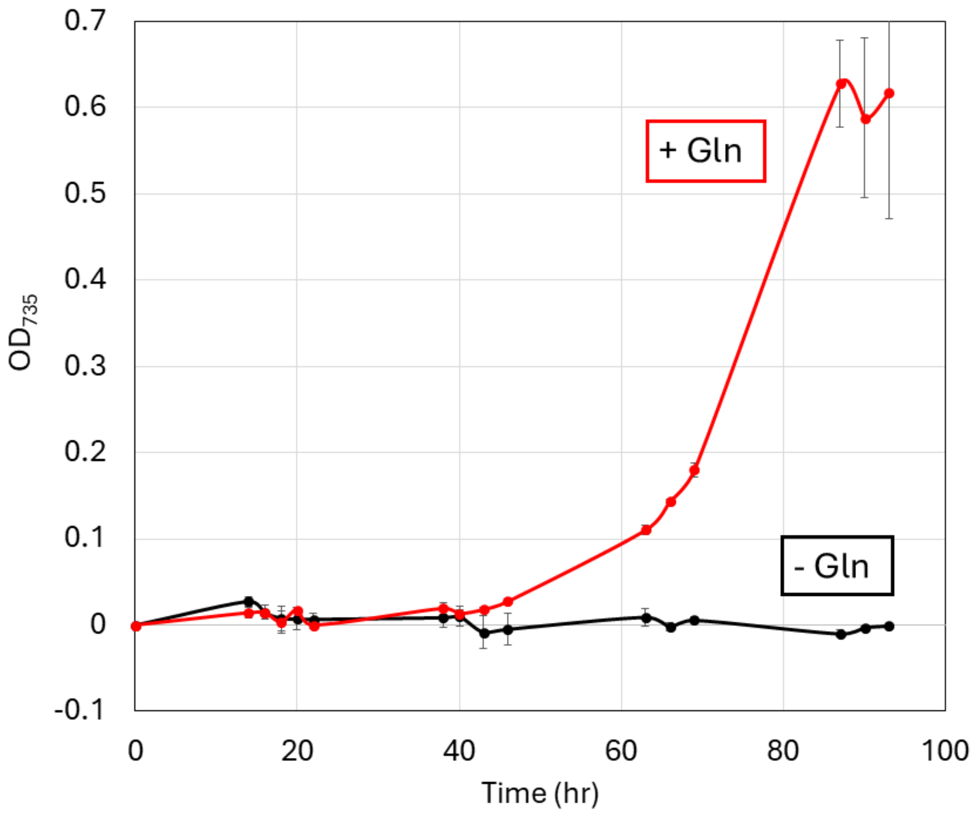

Figure S9. Growth of CS KO strain (95R-11) in acetate bicarbonate minimal media (ABMS) and formate with (+gln, red) and without (-gln, black) the addition of 10 mM glutamine supplement. Cultures were grown in 96-well plates with continual access to the $H_2/CO_2/N_2$ atmosphere of the anaerobic chamber. These data were collected before the strain was weaned off of glutamine. Points represent the average of three separately grown samples of 95R-11, and error bars represent the standard deviation (n=3). Lines do not represent fittings.
